# *RsaI *repetitive DNA in Buffalo *Bubalus bubalis *representing retrotransposons, conserved in bovids, are part of the functional genes

**DOI:** 10.1186/1471-2164-12-338

**Published:** 2011-07-01

**Authors:** Deepali Pathak, Sher Ali

**Affiliations:** 1 Molecular Genetics Laboratory, National Institute of Immunology, Aruna Asaf Ali Marg, New Delhi -110 067, India

## Abstract

**Background:**

Repetitive sequences are the major components of the eukaryotic genomes. Association of these repeats with transcribing sequences and their regulation in buffalo *Bubalus bubalis *has remained largely unresolved.

**Results:**

We cloned and sequenced *RsaI *repeat fragments pDp1, pDp2, pDp3, pDp4 of 1331, 651, 603 and 339 base pairs, respectively from the buffalo, *Bubalus bubalis*. Upon characterization, these fragments were found to represent retrotransposons and part of some functional genes. The resultant clones showed cross hybridization only with buffalo, cattle, goat and sheep genomic DNA. Real Time PCR, detected ~2 × 10^4 ^copies of pDp1, ~ 3000 copies of pDp2 and pDp3 and ~ 1000 of pDp4 in buffalo, cattle, goat and sheep genomes, respectively. *RsaI *repeats are transcriptionally active in somatic tissues and spermatozoa. Accordingly, pDp1 showed maximum expression in lung, pDp2 and pDp3 both in Kidney, and pDp4 in ovary. Fluorescence *in situ *hybridization showed repeats to be distributed all across the chromosomes.

**Conclusions:**

The data suggest that *RsaI *repeats have been incorporated into the exonic regions of various transcribing genes, possibly contributing towards the architecture and evolution of the buffalo and related genomes. Prospects of our present work in the context of comparative and functional genomics are highlighted.

## Background

Different families of repetitive DNA contribute towards architectural organization of the mammalian genomes [1]. They represent both, tandemly arranged and interspersed sequences [2]. Based on their size and mode of propagation, Interspersed elements can be divided into two separate classes, the long terminal repeat (LTR) and non-LTR. The non LTR LINEs (long interspersed repeat elements) and SINEs (Short interspersed repeat elements) are widely distributed occupying a substantial fraction of the eukaryotic genomes. These elements replicate and proliferate themselves through a "copy and paste" mechanism called retrotransposition [3,4]. In this process, transcription of their genomic copies is followed by an RNA intermediate resulting cDNAs reintegration at a new location in the genome [5]. Approximately, 100 LINE and SINE families have been reported to date in various eukaryotic genomes [6]. In mammals, LINE, L1 repeats are dominant retrotransposons type both in the common ancestor and in extant species [7]. In addition to L1, an element belonging to the retrotransposable element family of autonomous retrotransposons (RTE-1) has been reported in mammals [8]. Few mammals have active non LTR LINE other than L1 that contribute significantly to repeat composition.

Species specific retrotransposons have been widely used as a tool for Phylogenetic analysis and population studies [9,10]. Many retrotransposons are inactive, found in the non-coding regions of the genome and are subjected only to the neutral evolution. Thus, rare new insertions have led to some form of advantageous or noteworthy phenotypic variations [11-13]. These and other such discoveries have resulted in a shift from earlier thought of them being "parasite" to functional elements cultivated in the genome for their beneficial attributes.

Retrotransposons have gained novel functions, providing alternative splice sites and/or polyadenylation signals or modifying gene expression [14-16]. These elements account for 46.5% of bovine genome [17]. The bovine genome is very different in its repeat composition compared to other mammalian genomes. It has unusual composition of LINE RTE type Bov B and its associated SINE elements which together account for 25% of the bovine genome [17]. The impact of the interspersed repeats on the genomes of human [18-20], dog [21,22], cow [17,23], mouse [24] and opossum [25,26] has been studied. However fate of these interspersed elements and their association with mRNA transcriptomes in buffalo remains still unclear. Here, we report *RsaI *family repetitive DNA in the genome of water buffalo "*Bubalus bubalis*" and their copy number status. We also studied their expression in somatic tissues and spermatozoa. The repeat fraction pDp1, pDp2 and pDp3 were used for fluorescence *in situ *hybridization (FISH) with buffalo metaphase chromosomes. In addition, we isolated and sequenced *ACOT11 (*Acyl-coenzyme A thioesterase 11) gene harboring part of pDp1 repeat.

## Results

### *RsaI *enzyme digestion uncovers four repeat fractions

**Figure 1:**
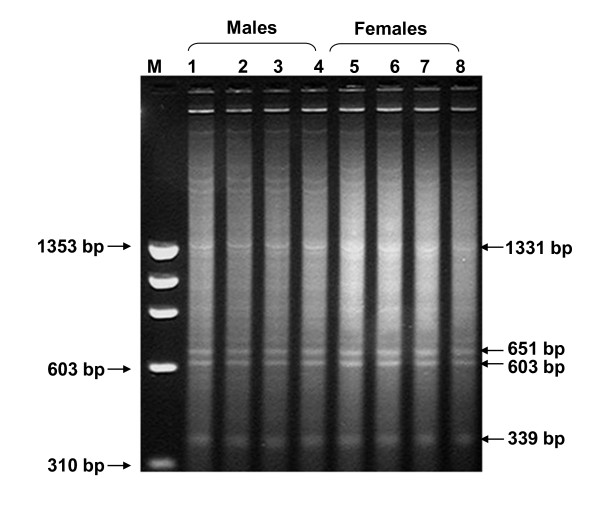
**Agarose gel showing restriction digestion of buffalo *Bubalus bubalis*, genomic DNA with *RsaI *enzyme**. The four discernible bands 1331, 651, 603 and 339 bp are highlighted. Molecular weight marker is given on the left in base-pair (bp).

Digestion of buffalo genomic DNA with *RsaI *enzyme, besides minor ones, showed four prominent bands ranging from 1331 base pairs, pDp1; 651, pDp2; 603, pDp3; to 339; pDp4 (Figure 1). Approximately, 15-20 recombinant clones subjected to restriction digestion and slot blot hybridization screening yielded ten positive clones for each repeat. Five-six clones from each fragment were then sequenced. The accession numbers of the recombinant clones are given in the Additional File 1. All the four major repeat elements were AT rich but sequence-wise, were different from one another (Additional File 2). No inter-clonal variations were detected in these sequences. However, random repeats were present in the nucleotide sequences (Table 1). Repeat Masker programme revealed presence of LTR LINE, SINE elements within the four fragments (Table 2). Blast search for each clone showed 69-98% homology with genomic DNA/contigs and 68-93% with transcribing genes in the database (Additional File 1) mostly in UTRs (Additional File 3).

**Table 1:** Details of repeat clusters present in *RsaI *sequences identified by repeat finder http://zlab.bu.edu/repfind/form.html

S. No.	Sequence ID	Repeat cluster	Location
1.	pDp1	CGTGAAC	1026,1033
		
		ATGTGAA	485, 666, 721, 734
		
		GTG	23, 41, 69, 161, 190, 198, 487, 668, 723, 736, 746, 780, 786, 800, 817, 832, 838, 879, 988, 1027, 1034, 1200
		
		GTGA	163, 198, 487, 668, 723, 736, 786, 800, 817, 832, 988, 1027, 1034
		
		GTGAA	487, 668, 723, 736, 817, 832, 1027, 1034
		
		TGTGAA	486, 667, 722, 735, 831,
		
		CGTGAA	816, 1026, 1033,
		
		GGATTTT	140,147
		
2.	pDp2	AAACTATGG	209, 375,592
		
		AAACTAT	209, 375, 468, 592
		
		ATACATTTGT	498, 538
		
3.	pDp3	AACATT	469, 481, 488
		
		TAT	15, 123, 139, 446, 451, 454, 464, 474, 583

**Table 2:** Genome-wide distribution of *RsaI *repeat fragments and their homologies with SINE and LINEs

S.No.	Sequence ID	GC level	Type of Elements	Subclass	Position of repeat in sequence	Length occupied (bp)	Percentage of the sequence in the buffalo genome
1.	pDp1	35.46	LINE2	L2	34-220	501	37.64
			
			LINE1	L1MEc	222-722	187	14.05
			
			RTE	BovB	730-785	532	39.97
			
				BTLTR	786-855		
			
				BovB	856-1331		
			
2.	pDp2	38.25	LINE 1	L1MC1s	9-651	643	98.77
			
3.	pDp3	39.47	LTR Elements (ERV_class I)	MER67C	7-212	206	34.16
			
4.	pDp4	30.68	SINE	BovB	1-159	44	12.98
			
			LINE (RTE)	BovA	160-203	159	46.90
			
			Low complexity	AT rich	245-310	66	19.47

### Buffalo derived *RsaI *Open Reading Frame (ORFs) has amino acid similarity to LINE reverse transcriptase

**Figure 2:**
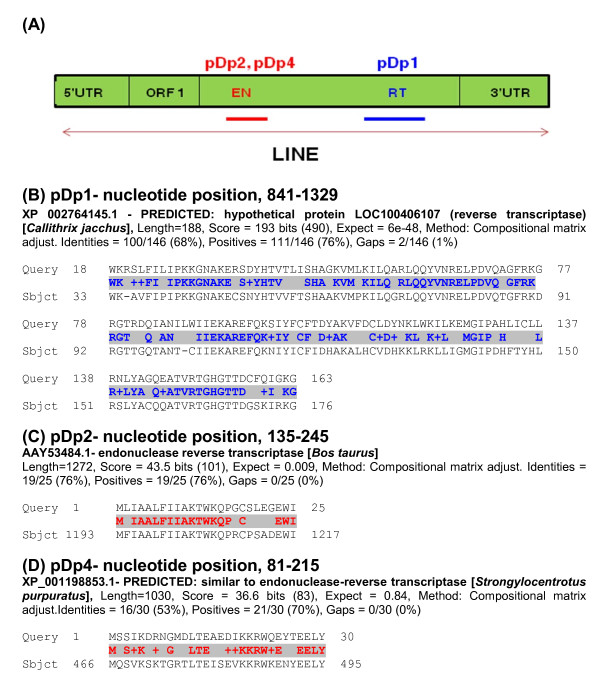
**Amino acid alignment of buffalo derived *RsaI *sequences showing positions of pDp1, 2 and 4 based on ORF search**. Panel **(A) **is a diagrammatic illustration showing 5'UTR, ORF1, Endonuclease, Reverse transcriptase and 3'UTS of LINE. Position of pDp1 (Blue), pDp2 and pDp4 (Red) within LINE are shown on top of the figure. The aligned sequences in the panel **(B) **shows region with homology to reverse transcriptase domain (Blue). Panel **(C) **and (**D)**, shows region with homology to endonuclease domain (Red).

**Figure 3:**
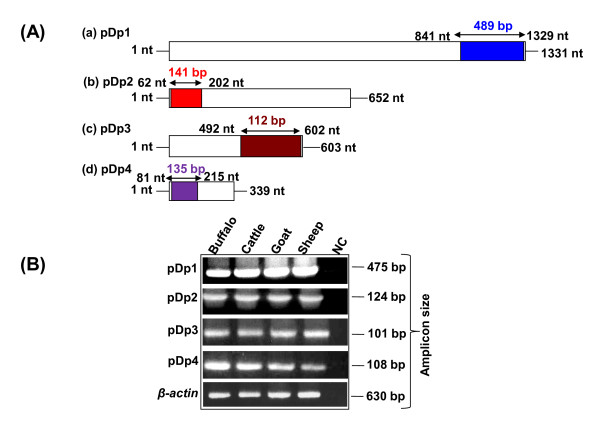
**PCR amplification of bovine genomic DNA using internal primers designed from the ORF of buffalo *RsaI *sequences**. Schematic representation **(A) **shows PCR strategy used for amplification of ORF regions of *RsaI *sequences corresponding to 489, 141, 112 and 135 bp, respectively **(a-d)**. Panel **(B) **shows PCR amplification of *RsaI *ORF regions and β-actin as control. The corresponding position of each PCR product is shown in Panel **(A)**. Sequence IDs are indicated on left, amplicons size on right and species are mentioned on top of the lanes.

Most striking feature of pDp1 sequence was the presence of 489 bp, +1 ORF (nucleotide position, 841-1329) (Figure 2B). BLASTP search with GenBank sequences using conceptual translation of this ORF (162aa) gave matches to putative reverse transcriptase's domain. This region corresponds to central position of the Reverse transcriptase ORF (Figures 2A and 3). Similarly, pDp2, 111 bp, +3 ORF (nucleotide position, 135-245, 65aa) and pDp4, 135 bp, +3 ORF (nucleotide position, 81-215, 44aa) showed homology to endonuclease reverse transcriptase (Figures 2B and 2C). These ORFs corresponded to central endonuclease reverse transcriptase of LINE1 ORF2 (Figure 3). pDp3 sequence,112 bp, +3 ORF (nucleotide position, 492-602, 37 aa) showed no similarity with endonuclease reverse transcriptase.

### *RsaI *Repeat status among bovids

Independent cross hybridization of pDp1, pDp2, pDp3 and pDp4 with genomic DNA from different species (mentioned in methods section) under high stringent conditions showed signals only in bovids (Additional File 4). PCR conducted using region specific primers (Table 3) amplified bands in buffalo, cattle, goat and sheep genomic DNA (Figures 3A &3B). Southern hybridization of two representative *RsaI *sequence pDp1, and pDp2 showed same number of bands within the genomic DNA of bovids (Additional File 5). Prominent bands within the smear reflect identical size of the fragments dispersed throughout the bovid genomes. ClustalW alignment of 489 bp pDp1derived fragment with cattle (AF060172), goat (AF404302) and sheep (AC148038) showed high level of sequence conservation among the bovids (Additional File 6). Phylogenetically, with respect to all the four sequences, cattle and buffalo were found to be closer to each other (Additional File 7), constituting the same monophyletic group.

**Table 3:** Details of the primers used for PCR amplification (A) Copy number and Relative expression studies by Real time PCR (B) in buffalo *Bubalus bubalis*

S.No.	Primer/Sequence ID	Forward	Reverse	Amplicon size (bp)	Annealing Temp (°C)	Nucleotide position of the primers in the sequence
		**(A) PCR Primers**				

1.	pDp1	TGCACTCAATATGCCAGAAAA	TGAAAGCAGTCTGTTGTTCCA	475	60	842-862 1296-1316

2.	pDp2	TGCAGCAATCACAATCCTTG	AACATCCTGGTTGCTTCCAA	124	60	66-85 170-189

3.	pDp3	TTGCACAGAATGAGCAGTGGT	GGCATTAATACTGGGATCAGG	101	60	492-512 572-592

4.	pDp4	GGACAGAAATGGTATGGACCT	TATATTGGAGAAGGCGATGGC	108	60	95-115 182-202

5.	*BBACOT11***(a)**	GAGCCGGCTGTCAGAGTC	AGCCAGACAACCTGGAATGT	2504	60	34-51 2518-2537

6.	*BBACOT11***(b)**	TGAAGCTGAAGCCAACTGTG	TCCTAAAAAGATCTTCATGACCA	2018	60	575-592 2570-2592

7.	*BBACOT11g*	AGTGAGGGGGTGTGCCCTGG	TGGTCGCACCCTCCTCGGAG	601	60	1-20 582-601

8.	*β-Actin*	CAGATCATGTTCGAGACCTTCAA	GATGATCTTGATCTTCATTGTGCTG	630	60	390-412 995-1019

9.	*CD45*	GACATCGCAGTGTTTGTTGC	GGAGGTTCACATTCCTCTCG	239	60	46-65 265-284

10	*CDH1*	TCTACAGCATCACTGGCCAACGAGCTG	TGCTTGGACCATCAGGGTGTATGTGGG	476	60	566-592 1015-1041

		**(B) Real Time PCR Primers**				

11.	Dp1	CCTGATGTTCAAGCTGGTTTTAGA	CAGAGGATGTTGGCAATTTGATC	65	60	1042-1065 1084-1106

12.	Dp2	GCAGCAATCACAATCCTTGGA	TGTAGGTTTCTGTGTGGGCATAA	63	60	67-87 107-129

13.	Dp3	CAAAGCAGTGCACAAGAATCAAT	TGCCTTGCCCAACCTTTTTA	63	60	318-340 361-380

14.	Dp4	AAGACCAGGGATCTCTTCAAGAAA	CGAGCTCATCTTTGCATGAAAT	66	60	24-47 68-89

15.	*GAPDH*	GCAAGTTCCACGGCACAGT	GATGGTGATGGCCTTTCCAT	68	60	227-245 275-294

### Differential expression of *RsaI *fragments in somatic tissue and spermatozoa of buffalo

**Figure 4:**
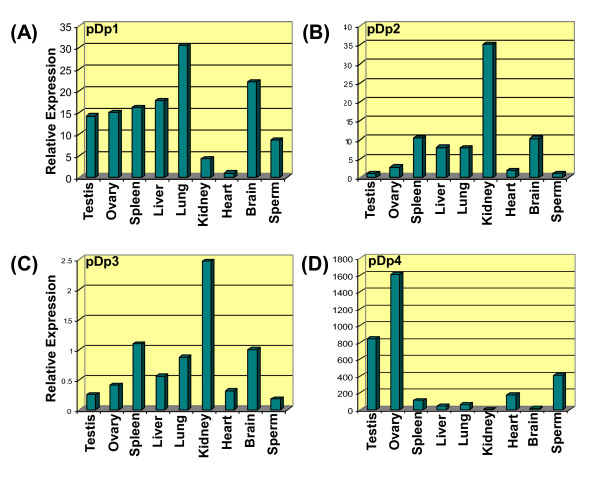
**Bar diagram based on the Real Time PCR amplification plot showing relative expression of pDp1, pDp2, pDp3 and pDp4 sequence, in different somatic tissues and spermatozoa (A-D)**.

RT-PCR analysis using internal primers of pDp1, pDp2, pDp3 and pDp4 (see Table 3) and cDNA from somatic tissues and spermatozoa of buffalo showed amplification of band across tissues and sperm confirming their transcriptional potentials (Additional File 8). Quantitative Real Time PCR analysis of these sequences showed differential expression of *RsaI *related transcripts across somatic tissues and spermatozoa (Figure 4). pDp1 showed highest expression in lung, pDp2 and pDp3 in Kidney and pDp4 in ovary. Summary of the relative expression (in folds) derived from 2^-ΔΔ*Ct *^values obtained for various transcripts based on Real Time PCR are given in Table 4.

**Table 4:** Relative expression analysis of the representative *Rsa*I mRNA transcripts in different somatic tissues and spermatozoa of buffalo *Bubalus bubalis*

S.No.	Transcript ID	Relative Expression (in folds) 2^-ΔΔ*Ct*^								
		
		Testis	Ovary	Spleen	Liver	Lung	Kidney	Heart	Brain	Sperm
1.	pDp1	14.25	15.02	16.11	17.68	30.49	4.34	*Cb*	22.06	28.62

2.	pDp2	0.99	2.77	10.36	7.88	7.83	10.29	35.09	1.84	*Cb*

3.	pDp3	1.50	44.01	10.30	57.48	18.37	*Cb*	5.20	1.35	6.56

4.	pDp4	841.41	1606.828	107.75	39.39	59.71	16.33	*Cb*	177.29	404.50

*Cb*-calibrator

### Multiple copies of *RsaI *fragments in bovids

The pair of internal primers deduced from pDp1-4 used for expression study was also employed for copy number analysis of these fragments in cattle buffalo, goat and sheep using Real Time PCR. Standard curve slope value was between 3.2-3.5. Single melting peak on dissociate curve confirmed primer specificity. pDp1 showed ~2 × 10^4 ^copies, pDp2 and pDp3, ~ 3000 copies each and pDp4 showed ~ 1000, in these species (Table 5 and Additional File 9).

**Table 5:** Absolute quantification of pDp1, pDp2, pDp3 and pDp4 copy number in buffalo, cattle, goat and sheep genomes

S. No.	Sequence ID	Species	Ct	Absolute quantity	Copies perhaploid genome
1.	pDp1	Buffalo	19. 89 ± 0.01		
				
		Cattle	19.89 ± 0.01	~ 6.0 × 10^5^	~ 2 × 10^4^
				
		Goat	19.89 ± 0.01		
				
		Sheep	19.85 ± .01		

2.	pDp2	Buffalo	28.71 ± 0.01		
				
		Cattle	28.71 ± .01	~ 457 × 10^4^	~ 3000
				
		Goat	28.70 ± 0.01		
				
		Sheep	28.69 ± 0.01		

3.	pDp3	Buffalo	30.30 ± 0.02		
				
		Cattle	30.18 ± 0.02	~ 448 × 10^4^	~ 3000
				
		Goat	30.18 ± 0.02		
				
		Sheep	30.30 ± 0.02		

4.	pDp4	Buffalo	32.09 ± 0.02		
				
		Cattle	32.0 ± 0.02	~ 308 × 10^3^	~ 1000
				
		Goat	32.09 ± 0.02		
				
		Sheep	32.06 ± 0.02		

Average ± standard deviation

### pDp1, pDp2 and pDp3 sequences are dispersed throughout the buffalo genome

**Figure 5:**
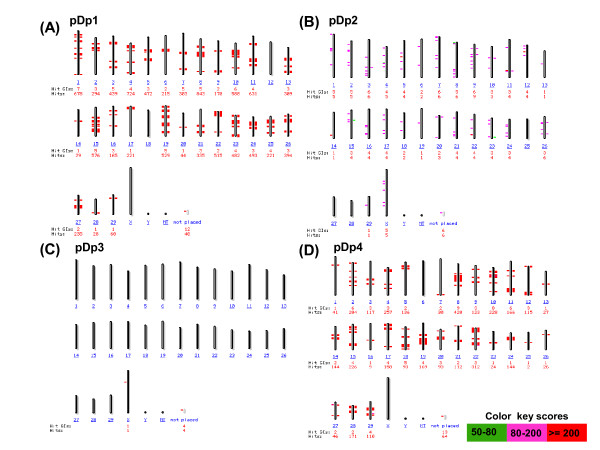
***In silico *mapping of *RsaI *sequences, pDp1 (A), pDp2 (B) pDp3 (C) and pDp4 (D) using existing cattle genomic map data (cow build 4 genome database)**. Note presence of pDp1, pDp2 and pDp4 sequences at multiple locations on cattle chromosomes. Color bar having percent homology of *RsaI *sequences with cattle genome is shown on right side of the figure.

**Figure 6:**
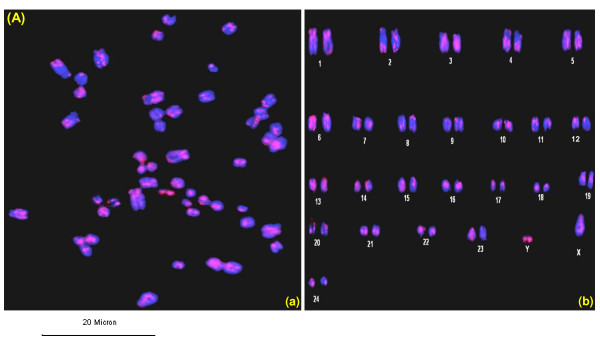
**Fluorescence *in situ *hybridization (FISH) of pDp1 (A) clone on buffalo metaphase chromosomes (a) and karyotype (b)**. Note the dispersed signals over the metaphase chromosomes. Scale bar used is given below the figure.

**Figure 7:**
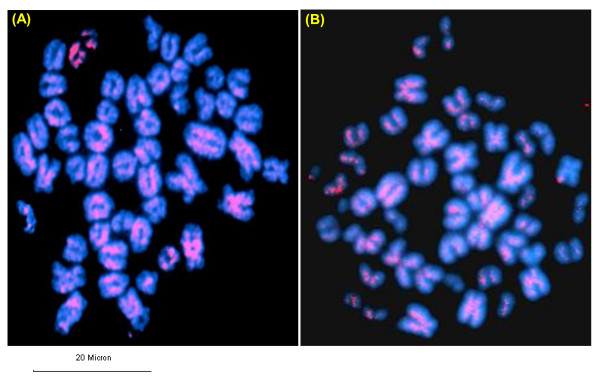
**Fluorescence *in situ *hybridization (FISH) of pDp2 (A) and pDp3 (B) clones onto buffalo metaphase chromosomes**. Note the dispersed signals over the chromosome arms. Scale bar used is given below the figure.

*In silico *analysis of pDp1, pDp2, pDp3 and pDp4 sequences using reference cattle genome revealed its multiple locations on the cow chromosomes (Figure 5). Probing of pDp1 to buffalo genomic DNA digested with *RsaI *enzyme detected a strong hybridization signal (Additional File 10) giving rise to a single isomorphic band in all the samples. FISH mapping of pDp1, 2, 3 spectrum red labeled cloned probes showed ubiquitous discernible signals over buffalo metaphase chromosomes (Figures 6 and 7). All the chromosomes showed dispersed pattern with all the three probes used independently. In several chromosomes, signals in the centromeric regions were absent or reduced giving rise to inconsistent pattern. FISH with pDp1 sequence showed more localized signals on the metaphase chromosomes as compared to that detected by pDp2 and pDp3. FISH with spectrum red labeled clone pDp4 probe showed background signals even after washing the slides under high stringent conditions (60°C in 0.1× SSC). This might be due to the short length (339 bp) of the probe pDp4 used coupled with its dispersed genomic organization.

### Full length cDNA sequence of *ACOT11 *gene in Buffalo

Blast search using reference mRNA sequence revealed *RsaI *repeats to be part of Acyl-coenzyme A thioesterase 11(*ACOT11*), *V*acuolar Protein Sorting 24 (*VPS24*) and Solute carrier organic anion transporter family member 1A2 (*SLCO1A2*) genes (Additional File 1) Full length Buffalo *ACOT11 *cDNA was generated using end point PCR and gene specific (*B. taurus ACOT11*) primers (Table 3). Assembled cDNA sequence of 2592 base pair fragment lacking poly A tail representing six exons (Additional Files 11 and 12) were deposited in the GenBank (HQ848649 and HQ848650).

## Discussion

We have studied four (pDp1, pDp2, pDp3 and pDp4) *RsaI *fragments from the buffalo genome which are AT rich, though buffalo genome on the whole is GC rich (40.69%, NC_006295). Database searches with the repeat-maskers revealed the presence of several LTR, LINE and SINE element in the four sequences (Table 2). Apparently, SINEs occupy the (G+C)-rich regions while LINEs are mainly located on the (A+T)-rich regions. Reports suggest that very large number of highly truncated insertions of L1 have occurred in the bovine genome [8]. Full length copies of the human L1 contain two open reading frames, ORF1 and 2. ORF1 encodes a DNA binding protein and ORF2 includes endonuclease and reverse transcriptase domains [27]. Presence of partial reverse transcriptase and endonuclease domains in pDp1, pDp2 and pDp4 reported herein led to the hypothesis that *RsaI *repeats might be related to a novel retrotransposable element.

Interspersed repeats get inserted into a new genomic location through the process of retrotransposition [28,29]. This is reflected by our FISH results of pDp1, 2 and 3 showing signals on all over the chromosomes with varying intensity. *In silico *analysis of pDp3 on the reference cattle genome showed fewer distribution suggesting its poor characterization in the cattle genome. This is supported by the fact that using real time PCR, we detected similar copy number of pDp3 in the bovids. Repetitive sequences in centromeric regions are dynamic components, ever prone to mutation, recombination, deletion, and translocation leading eventually to their alterations [30]. Absence of *RsaI *sequences in the centromere of several chromosomes may be undergoing such events. All the four sequences showed no homology with each other as revealed by clustalW alignment (Additional File 2). Startlingly, these four repeat elements were not detected in any of the non-bovid species. This suggests that irrespective of their origin and biological significance, their evolutions have been confined to limited number of species. Most likely, in non-bovid species, they were not favored evolutionarily and therefore purged slowly and gradually in due course of time. We presume that amplifications of repeat elements could have originated from its discrete blocks. Reintegration of extra chromosomal copies of these repeat elements [31] could have allowed its further dissemination in the genome. However, it is not clear whether all the repeats have similar significance in the buffalo genome. It is likely that these repeats are collectively involved in the evolution and sustenance of bovid chromosomes. Reports suggest that new retrotransposons are conserved within the same group of species [32]. This is corroborated by our Slot blot and PCR results. Real time PCR results showed approximately similar copy numbers for pDp1, 2, 3 and 4 in cattle, goat and sheep genomes as mentioned earlier. The copy number assessment of these repeats in different known and non-descript breeds of buffalo may enable to establish a correlation, if any, towards the delineation of different breeds.

Retrotransposons copies are reportedly involved in the regulation of transcription [33-36]. Presence of *RsaI *repeats in exonic database of various transcribing genes suggests that these sequences function as parts of mRNA. Since repeat sequences were also present in the introns, it is possible that they are transcribed as pre-mRNA and contribute to the processing of mRNA and splicing. Thus, differential expression of transcripts in somatic tissue and spermatozoa might be under the influence of post transcriptional regulation required for various cellular processes. Presence of transcribing retroelements within the buffalo spermatozoa reported here seems to be first such observation. Studies have shown existence of an RT-dependent mechanism operating in the spermatozoa, responsible for the genesis of new biologically active retrogenes [37]. These retrogenes may be delivered to the embryos during fertilization and propagated subsequently in the tissues of adult individuals [37].

*RsaI *elements were found to be part of the three functional genes *(ACOT11*, *VPS24 and SLCO1A2*) mostly present in 3' UTR. Our work corroborates recent reports that most part of retrotransposons inserts themselves in first and last exons and in untranslated regions (UTRs) [38]. In human, this type of insertion has been shown to create new non-conserved polyadenylation signals [39], influencing the level of gene expression [40]. However, how these insertions affect expression of buffalo transcriptomes is still a matter of speculation.

## Conclusions

Buffalo has several known and non-descript breeds of which a few are considered to be superior with respect to productivity and economic return. Whether, *RsaI *repeat in different breeds of buffalo would show similar organization and expression pattern is not known. However, if informative in breed delineation, these would prove to be useful biomarkers.

## Methods

### Species sample and DNA extraction

Approximately, 10 ml blood samples from both the sexes of buffalo, goat and sheep were collected from local slaughterhouse, Delhi following the guidelines of institute's Ethical and Biosafety Committee. Cattle blood sample was procured from the owner of the animal. Blood samples of human, fish, bird, rat, jungle cat, bonnet monkey, were available from other projects in the lab. Tiger, Indian rhinoceros and leopard samples were obtained with due permission from the competent authorities of the state and union government of India following strictly the guidelines of the Institute's Ethical and Biosafety Committee. Genomic DNA was extracted according to standard phenol-chloroform procedure [41].

### Restriction digestion of buffalo genomic DNA, cloning and sequencing

Approximately, 5 μg of buffalo genomic DNA from both the sexes was digested with *RsaI *restriction enzyme following supplier's (NEB) specification. Fragments were separated on 1% agarose gel in 1× TBE. Distinct bands within the smear were sliced from the gel, purified and cloned into dephosphorylated pBluescript II SK+ vector (Stratagene, USA), using standard protocol [42]. Approximately, ten clones, representing each fragment, were screened with restriction enzyme *Xho*1*/Nde*1 for the presence of insert. Slot-blot hybridization was conducted using *RsaI *fragments as probe labeled by random priming (RediprimeTM II kit, Amersham Pharmacia biotech, USA). Finally, positive clones were selected for sequencing.

### *In silico *analysis

Multiple sequence alignment and Phylogenetic tree construction were carried out using ClustalW program. Blast search were performed with the sequences in GenBank using BLASTN program (http://blast.ncbi.nlm.nih.gov/Blast.cgi?PROGRAM=blastn&BLAST_PROGRAMS=megaBlast&PAGE_TYPE=BlastSearch&SHOW_DEFAULTS=on&LINK_LOC=blasthome version 2.2.18+) with default parameters. Repeats were calculated using Repeat masker http://www.repeatmasker.org/cgi-bin/WEBRepeatMasker. Clustered repeats were found out using http://zlab.bu.edu/repfind/form.html ORFs and amino acid sequence identification was done using http://www.ncbi.nlm.nih.gov/gorf/gorf.html Conserved domain was determined using site http://www.ncbi.nlm.nih.gov/Structure/cdd/wrpsb.cgi Cattle chromosome map was constructed using NCBI *Bos taurus *genome view.

### Buffalo genomic DNA analysis

For cross hybridization studies, approximately 500 ng heat denatured genomic DNA from 14 species each mentioned earlier were slot-blotted onto the nylon membrane (Amersham) along with cloned plasmid as positive control and 2× SSC as negative control following standard protocol [41]. For Southern hybridization, approximately, 4-5 μg of buffalo, cattle, goat and sheep genomic DNA were subjected to restriction digestion using *RsaI *enzyme following supplier's (NEB) specifications. The digested DNA was resolved on 1.5% agarose gel and transferred onto the nylon membrane (Amersham) following standard protocol [42]. Membranes were rinsed in 2× SSC, dried and UV cross-linked. Blots were hybridized at 60°C overnight with α-32P-dCTP labeled recombinant plasmid (25 ng) using random priming method (rediprimeTM II kit, Amersham Pharmacia biotech, USA). Washing of the membranes was done using standard protocols and signals were recorded by exposure of the blot to X-ray film [41].

### PCR

The extent of sequence conservation across bovid genomes was further determined by PCR analysis. Primers were designed from ORF using Primer 3 software (Table 3). 50 ng of genomic DNA from buffalo, cattle, goat and sheep were PCR amplified using reaction conditions 95°C for 5 minutes followed by 35 cycles each consisting 95°C for 1 minute, 60°C for 1 minute and 72°C for 1 minute, and final extension at 72°C for 10 minutes for all the four fragments. Amplified fragments were resolved on 1.5% agarose gel in 1% TAE buffer (Table 3).

### RNA isolation and synthesis of cDNA

Total RNA was extracted from testis, kidney, liver, spleen, lung, heart, ovary, brain and sperm using TRIzol (Molecular Research Center, Inc., Cincinnati, OH) following manufacturer's instructions [43,44]. Tissues were procured from the local slaughterhouse, Delhi. Fresh ejaculated semen samples from buffalo bulls were obtained from the animal farm, Lucknow, U.P., India, strictly following the guidelines of the Institutes Ethical and Biosafety Committee. To check the contamination of mRNA from the cells other than spermatozoa, RNA extractions from the sperms were tested by RT-PCR for both the *CDH1 *(E-cadherin) GenBank Accession no. AJ400864 and *CD45 *(tyrosine phosphatase) GenBank Accession no. NM_001002763[45]. Similarly, presence of DNA was ruled out by PCR using β-actin primers, GenBank accession no. DQ661647 (Table 3). Following this, approximately 10 μg of RNA from different tissues and spermatozoa was reverse transcribed into cDNA using commercially available high capacity cDNA RT kit (Applied Biosystems, USA). The success of cDNA synthesis was confirmed by 35 cycles of PCR amplification using buffalo derived β-actin primers.

### RT-PCR and Relative expression analysis

Expression analysis of pDp1, pDp2, pDp3 and pDp4 transcripts using 50 ng cDNA from different somatic tissues and spermatozoa of buffalo was done with sequence specific internal primers designed by Primer 3 software (Table 3). PCR conditions were same as that mentioned earlier. β-actin was used as positive control. Relative expression analysis for the four transcripts was done using SYBR Green and Real time PCR Sequence Detection System-7500 (ABI, USA). The primers specific to the sequence were designed using Primer Express Software V2.0 (ABI) (Table 3). Primers for the housekeeping gene *GAPDH *(Glyceraldehyde 3-phosphate dehydrogenase) GenBank Accession no. XR_083674.1 was used to normalize the values for each sample. The specificity of each primer pair and efficiency of the amplification were tested by assaying serial dilutions of the cDNA. Each reaction was performed in triplicates and the mean value was used for the analyses [46]. The cyclic conditions comprise 50°C for 2 min and 95°C for 10 min, followed by 40 cycles each of 95°C for 10 s and 60°C for 1 min. Each experiment was repeated three times to ensure consistency of the results. The expression level of the desired sequence in different tissues and spermatozoa was calculated in the form of 2^-ΔΔCt ^value http://www3.appliedbiosystems.com/cms/groups/mcb_support/documents/generaldocuments/cms_042176.pdf[46].

### Copy number calculation

Real-time qPCR assays were performed in a 25 μl reaction volume containing 12.5 μl 2× SYBR Green^® ^PCR Master Mix (Applied Biosystems, Foster City, CA, USA), genomic DNA (0.1, 1.0 and 5 ng), forward and reverse primers at final concentration of 100 μM. Copy number estimation for four *RsaI *fragments in buffalo, cattle, goat and sheep genomes were done using 10 fold dilutions series of recombinant plasmids in the range 30,00,00000 to 3 copies (assuming haploid genome of farm animals =3.3 pg, wt per base pair = 1.096 × 10^-21 ^gm). Reactions were performed in 96-well MicroAmp Optical Reaction Plates (Applied Biosystems) in triplicates using the Real time PCR Sequence Detection System-7000 (ABI, USA) and SYBR Green dye. Primers and assay conditions were similar to those used for Relative expression studies (Table 3). Reaction specificity was confirmed with melting curves analysis. The standard curve was prepared using 10 folds dilution series of the recombinant plasmids and buffalo, cattle, goat and sheep genomic DNA [45,46].

### Chromosome preparation and fluorescence in situ hybridization (FISH)

Approximately, 200 μl of the whole blood from buffalo was cultured for chromosome preparation following standard protocols [46]. FISH was conducted with spectrum red labeled pDp1, pDp2 and pDp3 and pDp4 cloned probes (Abbott Molecular) on the metaphase chromosomes using Nick Translation Kit, Abbott Molecular, (Illinois, USA). Hybridization was carried out in 20 μl volume containing 50% formamide, 10% Dextran sulphate, Cot 1 DNA and 2× SSC, pH 7 for 16 hours at 37°C in a moist chamber. Post hybridization washes were done in 2× SSC at 37°C (low stringent condition) and then at 60°C in 0.1× SSC (under high stringent condition). Slides were counterstained with DAPI, screened under Olympus Fluorescence Microscope (BX51) and images were captured with Olympus U-CMAD-2 CCD camera. Chromosome mapping was done following the International System for Chromosome Nomenclature (ISCND 2000) for Bovids [47].

### Generation of full length buffalo *ACOT11 *mRNA using endpoint PCR

Blast search with pDp1 sequences showed 91% homology with *B. taurus ACOT11 *gene from nucleotide position 730-1331 encompassing 602 bp. Full length buffalo *ACOT11 *mRNA was generated using primers designed from *B. taurus ACOT 11 *(Accession No. NM_001103275). Details of Primer sequences and product size are given in Table 3. PCR amplified products were cloned into and pGEMT-easy vector and sequenced. Finally, buffalo *ACOT11 *gene sequences were assembled and full length sequence was deposited in the GenBank.

## Abbreviations

CD45: Cluster of differentiation 45; CDH1: Cadherin-1; cDNA: complementary Deoxyribonucleic acid; Ct: Cycle threshold; DAPI: 4',6-diamidino-2-phenylindole; GAPDH: Glyceraldehyde 3-phosphate dehydrogenase; mRNA: Messenger ribonucleic acid; RT-PCR: Reverse transcriptase Polymerase chain reaction; RTE: Retrotransposable element; RT-PCR: Reverse Transcriptase-Polymerase Chain Reaction; TAE: Tris/Acetic acid/EDTA; TBE: Tris/Borate/EDTA; UTR: Untranslated region.

## Authors' contributions

DP carried out the experiments and *in-silico *analysis, interpreted the data, and wrote the manuscript. SA conceived and designed the study, interpreted the results and revised the manuscript critically. All the authors read and approved the final manuscript

## Supplementary Material

Additional file 1 **Details of the Blast search**. Details of the Blast search of *RsaI *derived repeat sequences of water buffalo *Bubalus Bubalis*. Click here for file

Additional file 2 **Details of ClustalW alignment**. ClustalW alignment of buffalo derived *RsaI *element pDp1, pDp2, pDp3 and pDp4, showing each one as separate entity. Click here for file

Additional file 3 **ClustalW alignment with transcribing genes**. ClustalW alignment of buffalo *RsaI *pDp1, pDp2 and pDp4 sequences with *Bos taurus *transcribing genes (**A) ***ACOT11 ***(B) ***VPS24 *and **(C) ***SLCO1A2*. Sequences highlighted in yellow indicate UTR. *RsaI *sequences are marked in blue. Click here for file

Additional file 4 **Details of Cross-hybridization studies**. Cross-hybridization of *RsaI *recombinant clones with genomic DNA of different species. Signals were detected only in buffalo, cattle, goat and sheep as shown herein. PC denotes positive control (recombinant plasmids). IDs of the sequences used for hybridization are mentioned on the left. Click here for file

Additional file 5 **Details of Southern blot hybridization across bovids**. Representative blots showing distribution of pDp1 **(A) **and pDp2 **(B) **in buffalo, cattle, goat and sheep genome by Southern blot hybridization. Note discernible bands of 1331 and 652 bp in these species. Click here for file

Additional file 6 **Details of pDp1 alignment across the species**. ClustalW nucleotide alignment of buffalo pDp1, 489 bp ORF sequence, with cattle, goat and sheep sequences. Note the close sequence homology among the bovids. Click here for file

Additional file 7 **Phylogenetic analysis**. Phylogram based on percent identity of pDp1, pDp2, pDp3 and pDp4 **(A-D) **sequence in different species showing close relationship of buffalo with cattle. Click here for file

Additional file 8 **Details of RT PCR**. RT-PCR analysis of *RsaI *repeat sequences using internal primers and cDNA from different somatic tissues and spermatozoa of buffalo, Sequence IDs are indicated on the left and tissues are mentioned on top of the lanes. *β-actin *was used a positive control. M denotes 100 base pair marker. Click here for file

Additional file 9 **Details of copy number calculation with Real time PCR**. Standard curve based on 10 fold dilution series of pDp1, pDp2, pDp3, pDp4 and genomic DNA from buffalo, cattle, goat and sheep showing the amplification plot **(a-d) **panel **(A)**, corresponding slopes of -3.3 to -3.5, panel (**B) **and a single dissociation peak, panel **(C)**, substantiating maximum efficiency of the PCR reaction and high specificity of the primers with target DNA. Arrow indicates genomic DNA from buffalo, cattle, goat and sheep. Click here for file

Additional file 10 **Southern hybridization with pDp1 clone**. Southern hybridization of *Bubalus bubalis RsaI *digested genomic DNA with pDp1 clone **(A)**. The strongest isomorphic band corresponds to 1331 bp, indicated by an arrow **(B)**. Click here for file

Additional file 11 **Status of Exons in *ACOT11 *gene**. Pictorial representation showing *Bos taurus ***(A) **and *Bubalus bubalis ACOT11 *gene **(B) **with their representative exons. Nucleotide position 730 to 1331 indicates region of pDp1 showing 92% homology to *Bos taurus ACOT11*. Full length sequence of *Bubalus bubalis ACOT11 *gene lacking poly A tail and exons are given in **(C)**. Click here for file

Additional file 12 **ClustalW alignment of *ACOT11 *gene**. ClustalW alignment of buffalo *ACOT11 *gene with cattle (NM_001103275.1). Note the high level of sequence homology (92%) between the two species. Click here for file
