# Supplementary material for: RsaI repetitive DNA in Buffalo Bubalus bubalis representing retrotransposons, conserved in bovids, are part of the functional genes
Source: BMC Genomics. 2011 Jul 1;12:338. doi: 10.1186/1471-2164-12-338 (PMC3149587; doi:10.1186/1471-2164-12-338)
Supplement: Additional file 1 — Details of the Blast search. Details of the Blast search of RsaI derived repeat sequences of water buffalo Bubalus Bubalis. [file 1471-2164-12-338-S1.PDF]

| S.No. | Clone ID/ Length in bp/Accession No. | Database Searched | E-value | Query coverage | Identity (%) | Nucleotide position of the query sequence | Accession no. / length (bp) of homologues in bp | Nucleotide position of uncovered sequence | Description |
|---|---|---|---|---|---|---|---|---|---|
| 1 | pDp1/1331/ AY940196.1 | Btau_4.0 (mega blast) | 0.0 | 45% | 95 | 730-1331 | NW_001494496.2\|Bt29_WGA2180_4 / 1798093 | 980194- 980797 | *Bos taurus* chromosome 29 genomic contig, reference assembly (based on Btau_4.0) Features flanking this part of subject sequence: 241560 bp at 5' side: polycomb protein EED 13727 bp at 3' side: phosphatidylinositol-binding clathrin assembly protein |
| | | | | | Sequence present on multiple chromosomes | | | | |
| | | Nucleotide collection (nr/nt) | 0.0 | 45% | 94 | 730-1331 | AY944236.1/ 207929 | 90253-90856<br>45229-45830<br>51286-51889<br>13678-13078<br>99923-100396<br>10283-10752<br>182753-183342<br>103392-102813<br>138913-138355<br>52505-52311 | *Bos taurus* prion preproprotein (PRNP) and prion-like protein doppel preproprotein (*PRND*) genes, complete cds; and *RASSF2* gene, complete sequence |
| | | | 0.0 | 45% | 94 | 730-1331 | AJ871176 / 171712 | 73112- 72510<br>59325- 59932<br>171411- 170808<br>38560- 37959<br>113334- 113906<br>122107- 122702<br>112853- 112458<br>116659- 116949<br>49549- 49264<br>4709- 4621 | *Bos taurus* *ABCG2* gene, *PKD2* gene and *SPP1* gene, clone RPCI42_5K14 |
| | | | 0.0 | 45% | 95 | 730-1331 | AY644517.5/ 243699 | 82863- 83466<br>190820- 191423<br>150408- 149813 | *Bos taurus* T cell receptor gamma cluster 1 (*TCRG1*) |

| | | | | | | | | | |
|---|---|---|---|---|---|---|---|---|---|
| | | | | | | | | 102622- 102152<br>183784- 184376<br>111170- 111029<br>152660- 152726 | gene, complete sequence |
| | | | 0.0 | 45% | 93 | 730-1331 | AF060172/1139 | 338-942 | *Bos taurus* repeat region |
| | | | 0.0 | 45% | 92 | 729-1331 | AC147842.3/ 139569 | 43134- 42530 | *Ovis aries* clone CH243-255B6, complete sequence |
| | | Reference mRNA sequences (refseq_rna) | 0.0 | 45% | 90 | 730-1331 | NM_001103275.1/ 2628 | 2532- 1926 | *Bos taurus* acyl-CoA thioesterase 11 (*ACOT11*), mRNA |
| | | | 2e-127 | 26% | 91 | 257-604 | ref\|NM_174654.2/ 3504 | 2900-3249 | *Bos taurus* solute carrier organic anion transporter family, member 1A2 (*SLCO1A2*), mRNA |
| 2. | pDp2/ 651/ AY873795.1 | Btau_4.0 (mega blast) | 0.0 | 99% | 97 | 2-651 | NW_001495040.2\|Bt5_WGA568_4/ 2175351 | 1545534-1544884 | *Bos taurus* chromosome 5 genomic contig, reference assembly (based on Btau_4.0). Features in this part of subject sequence: protein phosphatase 1H (PP2C domain containing) isoform 2 |
| | | | | | Sequence present on multiple chromosomes | | | | |
| | | Nucleotide collection (nr/nt) | 6e-69 | 68% | 78 | 36-484 | AC099791.2/ 239704 | 169998- 170458 | *Homo sapiens* chromosome 1 clone RP11-430G17, complete sequence |
| | | | 1e-61 | 67% | 77 | 39-477 | AC188548.5/ 190240 | 106992- 106547 | *Canis Familiaris* chromosome 12, |

| | | | | | | | | | |
|---|---|---|---|---|---|---|---|---|---|
| | | | | | | | | | clone XX-511E22, complete sequence |
| | | | 6e-21 | 45% | 69 | 117-413 | AF136741.1/ 530 | 214- 515 | *Bos taurus* L1MC1-like repeat region, complete sequence |
| | | | 3e-37 | 52% | 71 | 141-483 | FN432136.1/ 127420 | 66562-97170 | *Ovis aries* pitx3 gene for paired-like homeodomain 3, BAC clone CH243-315I22 |
| | | Reference mRNA sequences (refseq_rna) | 1e-37 | 43% | 77 | 109-394 | XM_0011384271/ 3182 | 3133- 2848 | PREDICTED:*Pan troglodytes* vacuolar protein sorting 24, transcript variant 3 (VPS24), mRNA |
| | | | 1e-37 | 43% | 77 | 109-394 | NM_016079.2/ 3174 | 3123-2838 | *Homo sapiens* vacuolar protein sorting 24 homolog (*S.cerevisiae*) (*VPS24)*, transcript variant 1, mRNA |
| 3. | pDp3/603/ DQ020282.1 | Btau_4.0 (mega blast) | 0.0 | 99% | 98 | 2-603 | NW_001504011.1\| BtUn_WGA36024/ 44357 | 35042-35643 | *Bos taurus* genomic contig, reference assembly (based on Btau_4.0 ChrUn.004.1271) |
| | | | 0.0 | 99% | 86 | 7-593 | NW_001508692.2\| BtX_WGA2236_4/ 391646 | 230121-229522 | *Bos taurus* chromosome X genomic contig, reference assembly (based on Btau_4.0) Features flanking this part of subject sequence: 36076 bp at 5' side: hypothetical protein LOC100302527 |

| | | | | | | | | | |
|---|---|---|---|---|---|---|---|---|---|
| | | | | | | | | | 54504 bp at 3' side: family with sequence similarity 127, member A-like |
| | | | 4e-<br>133 | 72% | 86 | 12-447 | ref\|NW_00310391<br>4.1/1620457 | 928476<br>-928039 | *Bos taurus* breed Hereford chromosome 5 genomic scaffold, alternate assembly Bos_taurus_UMD _3.1, whole genome shotgun sequence. features in this part of subject sequence: PRKC apoptosis WT1 regulator protein-like. |
| | | Reference mRNA sequences (refseq_rna) | 1e-04 | 10% | 79 | 125-187 | NR_026974<br>XR_041327<br>XR_041328/3250 | 1360-1423 | *Homo sapiens* chromosome 8 open reading frame 77 (C8orf77), non-coding RNA |
| 4. | pDp4/339/<br>DQ131586 | Btau_4.0<br>(mega blast) | 3e-80 | 50% | 98 | 1-170 | NW_001495461.2\|<br>Bt8_WGA920_4/<br>1476140 | 289076- 289246<br>703271- 703441<br>1269801-<br>1269631<br>1258485-<br>1258315<br>855919- 855749<br>1217125-<br>1217295<br>1210469-<br>1210639<br>396001- 396171<br>841365- 841535<br>1204853-<br>1204695<br>385942- 385780 | *Bos taurus* chromosome 8 genomic contig, reference assembly (based on Btau_4.0) |
| | Sequence present on multiple chromosomes | | | | | | | | |

| | | | | | | | | |
|---|---|---|---|---|---|---|---|---|
| | Nucleotide collection (nr/nt) | 1e-76 | 87% | 97 | 1-170 | AC149783.4/ 205084 | 110423- 110253<br>138412- 138245 | *Bos taurus* BAC CH240-3K17 (Children's Hospital Oakland Research Institute Bovine BAC Library complete sequence. |
| | | 7e-69 | 59% | 94 | 1-170<br>1-170<br>1-170<br>1-170<br>1-175<br>1-170<br>1-161<br>73-169<br>1-82<br>167-199<br>167-196<br>167-199<br>160-199<br>160-199<br>160-199<br>160-199<br>168-197<br>168-197<br>167-195<br>171-199<br>160-199 | AC148245.3/ 153498 | 126277- 126107<br>152716- 152546<br>77825- 77995<br>122902- 123072<br>2611- 2783<br>45960- 45791<br>10704- 10862<br>106525- 106621<br>105959- 106040<br>86492- 86524<br>6368- 6339<br>34943- 34911<br>6515- 6479<br>21672- 21636<br>86345- 86381<br>129399- 129435<br>24358- 24329<br>134277- 134248<br>26-94<br>95746- 95718<br>133200- 133236 | *Ovis aries* clone CH243-293L12, complete sequence |
| | Reference mRNA sequences (refseq_rna) | 1e-25 | 23% | 93 | 90-170 | NM_174654.2/ 3504 | 2761-2841 | *Bos taurus* solute carrier organic anion transporter family, member 1A2 (*SLCO1A2*), mRNA |

The “Percentage”, “E-value”, “Accession no.” and “Description” are taken from the highest relevant hit obtained from the BLAST query at NCBI database collection. The databases consulted are mRNA, genomic nucleotide or Btau_4.0 references.

# Additional File 1
