# Supplementary material for: RsaI repetitive DNA in Buffalo Bubalus bubalis representing retrotransposons, conserved in bovids, are part of the functional genes
Source: BMC Genomics. 2011 Jul 1;12:338. doi: 10.1186/1471-2164-12-338 (PMC3149587; doi:10.1186/1471-2164-12-338)
Supplement: Additional file 2 — Details of ClustalW alignment. ClustalW alignment of buffalo derived RsaI element pDp1, pDp2, pDp3 and pDp4, showing each one as separate entity. [file 1471-2164-12-338-S2.PDF]

SeqA	Name	Len (nt)	SeqB	Name	Len (nt)	Score
1	pDp1	1331	2	pDp2	651	6
1	pDp1	1331	3	pDp3	603	6
1	pDp1	1331	4	pDp4	339	17
2	pDp2	651	3	pDp3	603	4
2	pDp2	651	4	pDp4	339	5
3	pDp3	603	4	pDp4	339	7

```
pDp1            ACAAGCGCTTTAAGGGGAGCATGTGTTTGGTCAGAGGCCAGTGTGGCTGGGGAAGAATTA 60
pDp4            ------------------------------------------------------------
pDp3            ------------------------------------------------------------
pDp2            ------------------------------------------------------------


pDp1            AGATATGAGTGGGAGGAGACATTAAATCAGAAAGGTAACTTAGGATCAGATCATATAGAA 120
pDp4            ------------------------------------------------------------
pDp3            ------------------------------------------------------------
pDp2            ------------------------------------------------------------


pDp1            TGTAACAGATCATGGGTTAGGATTTTGGATTTTATATTAAGTGTGATTGGAATTCATTAG 180
pDp4            ------------------------------------------------------------
pDp3            ------------------------------------------------------------
pDp2            ------------------------------------------------------------


pDp1            AGAGTTTTTGTGGAGGAGTGACATTATCAGATTTATATCTGTAAAAGAAGATCTTCTGGC 240
pDp4            ------------------------------------------------------------
pDp3            ------------------------------------------------------------
pDp2            ------------------------------------------------------------


pDp1            AAAAGAAAAATTACAGCAGATGGAAATTTGAATCTATACAGAAAAATACAGAGCACTGGA 300
pDp4            ------------------------------------------------------------
pDp3            ------------------------------------------------------------
pDp2            ------------------------------------------------------------


pDp1            AATGGTAACTATGCGCTACATATAAAATACTTTATTTTCCTTGCATTTAAATGTCTCAAA 360
pDp4            ------------------------------------------------------------
pDp3            ------------------------------------------------------------
pDp2            ------------------------------------ATACCTTAACTTCAGAACACCACT 24


pDp1            AAGACAATTGACTCTAAAAAAAATAGAGAGTT--TATAACACATAGAGGCAAATCATATG 418
pDp4            ------------------------------------------------------------
pDp3            ---------CACTGCAGGGAAAATATAAAGTTA-CATAAGAAACAAAGTTTTAATTTTCC 50
pDp2            GAGTGATTTGCTACAAAACTGAACATACTCTTACCATATGATGCAGCAATCACAATCCTT 84


pDp1            TCAACAAAGGCACAAAGATAGGGAGAAGAAAAATAAAAGTATACTGCTATAAAATTATTA 478
pDp4            ------------------------------------------------------------
```

```
pDp3            CCATTGAAAACTGACAGCAGGCCTTCACCCCTCCCTTTGCTTTAGAGGGTTAACTTTGCA 110
pDp2            GGAATCCAGCATGGTTGAAAATTTATGCCCACACAGAAACCTACATATGGATGCTAATAG 144

pDp1            TA-TTACATGTGAAATGCTACAATATTAGTTGAAGGTAAATTGATAATTAAAGATATATA 537
pDp4            ------------------------------------------------------------
pDp3            AA-AGGTGTCAGTTATTTCTCTGCCTCT-TTATATGTGTAGAAATCATTAAAAAAGCTCA 168
pDp2            CAGCTTTATTCATAATTGCCAAAACTTGGAAGCAACCAGGATGTTCTTTAGAAGGTGAGT 204

pDp1            ATATAAACTATAAAAATCACTAAAA---TAACAAAATGAAAAAATATAGTTAATAA-GTC 593
pDp4            ---------ATAGAGG-----AAAA---CAACAGAATGGAAAGACCAGG------G-ATC 36
pDp3            ATAAGCCTCTTGCCAAT---TTGAA---TGCTCCTTT--AAGGACCTGGCAGGAAA-ATC 219
pDp2            GGATAAACTATGGTATATCCAGAAAACGTATTATCATTTAGTGCTAAAAAGAACTACAAG 264
                          *            **           *  *

pDp1            AGCAAAGGAGAAACAATGGAACCATA-ACACAATTAATTCAA-AAAAAGACCGAGAGAAG 651
pDp4            TCTTCAAGAAAATCAGAGATACCAAAGAAACATTTCATGCAA-AGATGAGCTCGATAAAG 95
pDp3            CTTGCAAAAGGGGAGAGCTAGGTAAGGAAAGAAAAACTTAAGCAGTTGAAACCAGAAACT 279
pDp2            CTATCAAAGGATGTGGAAGAATCTTAAATGCACGTTATTTAGTGAAAGAAGCC---AACC 321
                     *                     *   *     *  *                *

pDp1            GGAAAAAACAACAGATGTGAAAAATAGAAAACAAATAGCAATATGGTAGATTTAAACCCA 711
pDp4            GACAGAAATGGTATGGACCTAACAGAAGCAGAAGATATTAAGAAGAGATGGCAAGA---A 152
pDp3            GCTAGACCTTCTGGACACTCAGAGAAAGAAATGACCATCAAAGCAGTGCACAAGAATCAA 339
pDp2            TGAAAAGGTTGCATAT-TGAAGGATTCCAAGTAAATGACATTCTGAAAGAGGCAAAACTA 380
                   * *              *        *         *               *   *

pDp1            CTCA----TATCAATGTGAATTTGGAATGTGAAGTCAAGTGGGCT-TAGAAAGCATCACT 766
pDp4            TACA----CAGAAGAACTGTACTGGAATGGGGTGCCA--TCGCCT-T------CTCCAAT 199
pDp3            TAAAAGTTCAGGAGAAGATCATAAAAAGGTTGGGCAAGGCAGCATCTTAAGAGGATTACA 399
pDp2            TGGAGA--CAGTAAAAGATCACTGGTTGCCAGGGTCTAGAGGGGA------GAGAGCCAT 432
                   *     *  *                    *       *

pDp1            ATAAACAAAGCTAGTGGAGGTGATGGAATTCCAGTGAGCTATTCCATATCGTGAAAGATG 826
pDp4            ATATAGTTACATATAACAAATAAT----TTATAGT----TATACTATAT-GCAGTAAATA 250
pDp3            GCATCCACAGAAGCAGGAGATGTTCCTTCTCAGAACTTCCACAGGTTATGTTATTATGCA 459
pDp2            GAATCAGCAGAGCACAGAGGGTTTCTAGGGCAGTGAAACTATTCTGAATGACGTTACACA 492
                  *     *        *     *                *      **      *

pDp1            ATGCTGTGAAAGTG---CTGCACTCAATATGCCAGAAAATTTGGAAAACTCA-GCAGTGG 882
pDp4            ATATTGTAAAATAT---TTAAAT--AATAT-TAATAAATTTTATCTCACATT-ATAATAT 303
pDp3            ACAGTATAAAACA----TTATAAAGAACATTAAACATTGCACAGAATGAGCA-GTGGTAG 514
pDp2            CCATTATACATTTGTCATTATGTATAATGGTGGATACATGTCATGATACATTTGTTAAAA 552
                    * *  *        *       **      * *

pDp1            CCACAGGA----CTGGAAAAGGTCACTTTTCATT-CTAATCCCAAAGAAAGGCAATGCCA 937
pDp4            TTATA-------TTGAAAAATGGGGCTTC-CTTC-CTGAACTAAA--------------- 339
pDp3            CAAAATCATCTCCTGGCAGAGAGGCCCACAGTTCACTAGGAAAATGAAATGTGAAGACCT 574
pDp2            CTCAAAGAATGTGTAACACCAAGAGTGAATCCTC------AATGTAAACTATGGATTCCG 606
                    *        *   *              *

pDp1            AAGAACGCTCAGACTACCACACAGTTACACTCATCTCACACGCTGGTAAAGTGATGCTCA 997
pDp4            ------------------------------------------------------------
pDp3            GATCCCAGTATTAATGCCTGAATGTCAGT------------------------------- 603
pDp2            GATGATACTGGTGTGTCCAGGTAGCTTCATGGATTGTAGCGAATG--------------- 651

pDp1            AAATTCTCCAAGCCAGGCTTCAACAATACGTGAACCGTGAACTTCCTGATGTTCAAGCTG 1057
pDp4            ------------------------------------------------------------
pDp3            ------------------------------------------------------------
pDp2            ------------------------------------------------------------
```

```
pDp1            GTTTTAGAAAAGGCAGAGGAACCAGAGATCAAATTGCCAACATCCTCTGGATCATTGAAA 1117
pDp4            ------------------------------------------------------------
pDp3            ------------------------------------------------------------
pDp2            ------------------------------------------------------------

pDp1            AAGCAAGAGAGTTCCAGAAAAGCATATATTTCTGCTTTACTGACTATGCCAAAGTCTTTG 1177
pDp4            ------------------------------------------------------------
pDp3            ------------------------------------------------------------
pDp2            ------------------------------------------------------------

pDp1            ACTGTCTGGATTACAATAAACTGTGGAAAATTCTGAAAGAGATGGGAATACCAGCCCACC 1237
pDp4            ------------------------------------------------------------
pDp3            ------------------------------------------------------------
pDp2            ------------------------------------------------------------

pDp1            TGATCTGCCTCTTGAGAAATTTGTATGCAGGTCAGGAAGCAACAGTTAGAACTGGACATG 1297
pDp4            ------------------------------------------------------------
pDp3            ------------------------------------------------------------
pDp2            ------------------------------------------------------------

pDp1            GAACAACAGACTGCTTTCAAATAGGAAAAGGAGT 1331
pDp4            ----------------------------------
pDp3            ----------------------------------
pDp2            ----------------------------------
```

Additional File 2
