# Supplementary material for: RsaI repetitive DNA in Buffalo Bubalus bubalis representing retrotransposons, conserved in bovids, are part of the functional genes
Source: BMC Genomics. 2011 Jul 1;12:338. doi: 10.1186/1471-2164-12-338 (PMC3149587; doi:10.1186/1471-2164-12-338)
Supplement: Additional file 3 — ClustalW alignment with transcribing genes. ClustalW alignment of buffalo RsaI pDp1, pDp2 and pDp4 sequences with Bos taurus transcribing genes (A) ACOT11 (B) VPS24 and (C) SLCO1A2. Sequences highlighted in yellow indicate UTR. RsaI sequences are marked in blue. [file 1471-2164-12-338-S3.PDF]

(A)

```
pDp1            ACTCCTTTTCCTATTTGAAAGCAGTCTGTTGTTCCATGTCCAGTTCTAACTGTTGCTTCC 60
BTACOT11        ACTCCTTTTCCTATTTGGAACCAGTCTCTTGTTCCATGTCCAGTTCTAACTGTTGCTTCC 60
                ***************** ** ****** ********************************

pDp1            TGACCTGCATACAAATTTCTCAAGAGGCAGATCAGGTGGGCTGGTATTCCCATCTCTTTC 120
BTACOT11        TGACCTGCATATAGGTTTCTCAAGAGGCAGGTCAGGTGGTCTGATATTCCCATCTCTTTC 120
                *********** *  *************** ******** *** ****************

pDp1            AGAATTTTCCACAGTTTATTGTAATCCAGACAGTCAAAGACTTTGGCATAGTCAGTAAAG 180
BTACOT11        AGAATTTTCCACAGTTTCTTGTAATCCACACAGTCAAAGGCTTTGGCATAGTCGAGAAAG 180
                ***************** ********** ********** *************   ****

pDp1            CAGAAATATATGCTTTTCTGGAACTCTCTTGCTTTTTCAATGATCCAGAGGATGTTGGCA 240
BTACOT11        CAGAAATAGATGTTTTTCTTGAACTCTCTTGCTTTTTCCATGATCCAGTGGATGTTGGCA 240
                ******** *** ****** ****************** ********* ***********

pDp1            ATTTGATCTCTGGTTCCTCTGCCTTTTCTAAAACCAGCTTGAACATCAGGAAGTTCACGG 300
BTACOT11        ATTTCGTCTCTGGTTCCTCTGCCTTTTCTAAAACCAGTTTGAACATCAGGAAGTTCACGG 300
                ****  ******************************** *********************

pDp1            TTCACGTATTGTTGAAGCCTGGCTTGGAGAATTTTGAGCATCACTTTACCAGCGTGTGAG 360
BTACOT11        TTCACGTATTGCTGAAGCCTGGCTTGGAGAATTTTGAGCATTACTTTACTAGCGTGTGAG 360
                *********** ***************************** ******* **********

pDp1            ATGAGTGTAACTGTGTGGTAGTCTGAGCGTTCTTTGGCATTGCCTTTCTTTGGGATTAGA 420
BTACOT11        ATGAGTGCAATTGTGCAGTCGTTTGAGCATTCTTTGGCATTGCCTTTCTTTGGGATTGAA 420
                ******* ** ****  ** ** ***** ****************************  *

pDp1            ATGAAAAGTGACCTTTTCCAGTCCTGTGGCCACTGCTGAGTTTTCCAAATTTTCTGGCAT 480
BTACOT11        ATGAAAACTGACCTTTTCCAGTCCTGTGACTACTGCTGAGTTTTCCAAATGTGCTGGCAT 480
                ******* ******************** * ******************* * *******

pDp1            ATTGAGTGCAGCACTTTCACAGCATC---ATCTTTCACGATATGGAATAGCTCA-CTGGA 536
BTACOT11        ATTGAGTGCAGCACTTTCACAGCATCGTCATCTTCTAGGATTTGAAATAGTTCAACTGGA 540
                **************************   *****  * *** ** ***** *** *****

pDp1            ATTCCATCACCTCCACTAGCTTTGTTTATAGTGATGCTTTCTAAG-CCCACTTGACTTCA 595
BTACOT11        ATTCCATCACCTCCACTAGCTTTGTTTGTAGTGATGCTTTCTAAGGCCTACTTGACTTCA 600
                *************************** ***************** ** ***********

pDp1            CATTCCA 602
BTACOT11        CATTCCA 602
                 *******
```

AATTCACATTGATATGAGTGGGTTTAAATCTACCATATTGCTATTTGTTTTCTATTTTTCACATCTGTTGTTTTTTCCCTTCTCTC
GGTCTTTTTTTGAATTAATTGTGTTATGGTTCCATTGTTTCTCCTTTGCTGACTTATTAACTATATTTTTTCATTTTGTTATTTTA
GTGATTTTTATAGTTTATATTATATATCTTTAATTATCAATTTACCTTCAACTAATATTGTAGCATTTCACATGTAATATAATAAT
TTTATAGCAGTATACTTTTATTTTTCTTCTCCCTATCTTTGTGCCTTTGTTGACATATGATTTGCCTCTATGTGTTATAAACTCTC
TATTTTTTTTAGAGTCAATTGTCTTTTTGAGACATTTAAATGCAAGGAAAATAAAGTATTTTATATGTAGCGCATAGTTACCATTT
CCAGTGCTCTGTATTTTTCTGTATAGATTCAAATTTCCATCTGCTGTAATTTTTCTTTTGCCAGAAGATCTTCTTTTACAGATATA
AATCTGATAATGTCACTCCTCCACAAAAACTCTCTAATGAATTCCAATCACACTTAATATAAAATCCAAAATCCTAACCCATGATC
TGTTACATTCTATATGATCTGATCCTAAGTTACCTTTCTGATTTAATGTCTCCTCCCACTCATATCTTAATTCTTCCCCAGCCACA
CTGGCCTCTGACCAAACACATGCTCCCCTTAAAGCGCTTGT- 1331

(B)

```
pDp2           ATACCTTAACTTCAGAACACCACTGAGTGATTTGCTACAAAACTGAACATACTCTTACCA

pDp2           TATGATGCAGCAATCACAATCCTTGGAATCCAGCATGGTTGAAAATTTATGCCCACACAG 120
BTPTVPS24      ------------------------------------------------ATGCCCACAGAA 12
                                                               ********* * 

pDp2           AAACCTACATATGGATGCTAATAGCAGCTTTATTCATAATTGCCAAAACTTGGAAGCAAC 180
BTPTVPS24      AAACCTGCACAAAGATGTTTACAACAGCTTTATTCATAATTGCCAAAACTTGGAAACCAC 72
               ****** ** *  **** * * * ******************************* * **

pDp2           CAGGATGTTCTTTAGAAGGTGAGTGGATAAACTATGGTATATCCAGAAAACGTATTATCA 240
BTPTVPS24      CAAGAAGTCTTTCAATACGTGAATGGATAAACTA---TTCATCTAGACAATGGAGTATTA 129
               ** ** **  ** *  * **** ***********   *  *** *** ** * * *** *

pDp2           TTTAGTGCTAAAAAGAACTACA------AGCTATCAAAGGATGTGGAAGAATCTTAAATG 294
BTPTVPS24      TTCAGTGCTAAAAATAAATGCACTATTAAGCCATGAAATGACATGGAGGAACCTTAATTG 189
               ** *********** ** * **      *** ** *** **  **** *** ***** **

pDp2           CACGTTATTTAGTGAAAGAAGCCAACCTGAAAAGGTTGCATATTGAAGGATTCCAAGTAA 354
BTPTVPS24      CATATTATTAAGTGAAAGAAGCCAGTCTGAAAAGGCTACATACTATATGATTCCAAT--- 246
               **  ***** **************  ********* * **** *  * ********    

pDp2           ATGACATTCTGAAAGAGGCAAAACTATGGAGACAGTAAAAGATCACTGGTTGCCAGGGTC 414
BTPTVPS24      ATGACATTCTAGAAAAGGAAGAACTATGGCGACAGTAAAA-------------------- 286
               **********  ** *** * ******** **********                    

TAGAGGGGAGAGAGCCATGAATCAGCAGAGCACAGAGGGTTTCTAGGGCAGTGAAACTATTCTGAATGACGTTACACACCATTATA
CATTTGTCATTATGTATAATGGTGGATACATGTCATGATACATTTGTTAAAACTCAAAGAATGTGTAACACCAAGAGTGAATCCTC
AATGTAAACTATGGATTCCGGATGATACTGGTGTGTCCAGGTAGCTTCATGGATTGTAGCGAATG-651
```

(C)

```
pDp4           ATAGAGGAAAACAACAGAATGGAAAGACCAGGGATCTCTTCAAGAAAATCAGAGATACCA

pDp4           AAGAAACATTTCATGCAAAGATGAGCTCGATAAAGGACAGAAATGGTATGGACCTAACAG 120
BTSLCO1A2      -----------------------------ATAAAGGACAGAAATGGTATGGACCTAACTG 31
                                            ***************************** *

pDp4           AAGCAGAAGATATTAAGAAGAGATGGCAAGAATACACAGAAGAACTGTACTGGAATGGGG 180
BTSLCO1A2      AAGCAGAAGATATTGAGAAGAGGTGGCAAGAATACATAGAAGAACTATAC---------- 81
               ************** ******* ************* ********* ***          
TGCCATCGCCTTCTCCAATATATAGTTACATATAACAAATAATTTATAGTTATACTATATGCAGTAAATAATATTGTAAAATATTT
AAATAATATTAATAAATTTTATCTCACATTATAATATTTATATTGAAAAATGGGGCTTCCTTCCTGAACTAAA 339
```

Additional File 3
