# Supplementary figures and images for: RsaI repetitive DNA in Buffalo Bubalus bubalis representing retrotransposons, conserved in bovids, are part of the functional genes
Source: BMC Genomics. 2011 Jul 1;12:338. doi: 10.1186/1471-2164-12-338 (PMC3149587; doi:10.1186/1471-2164-12-338)

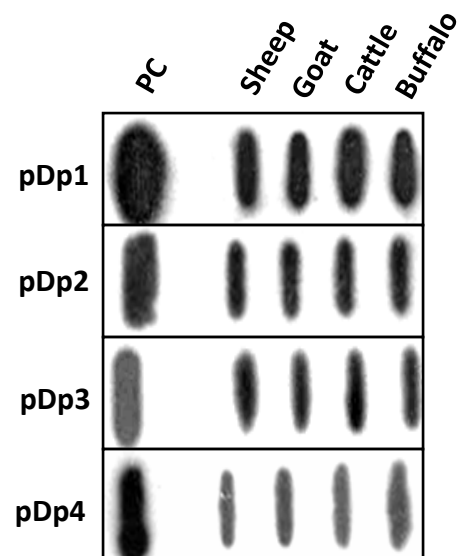

**Additional File 4**

Supplement: Additional file 4 — Details of Cross-hybridization studies. Cross-hybridization of RsaI recombinant clones with genomic DNA of different species. Signals were detected only in buffalo, cattle, goat and sheep as shown herein. PC denotes positive control (recombinant plasmids). IDs of the sequences used for hybridization are mentioned on the left. [file 1471-2164-12-338-S4.PDF]

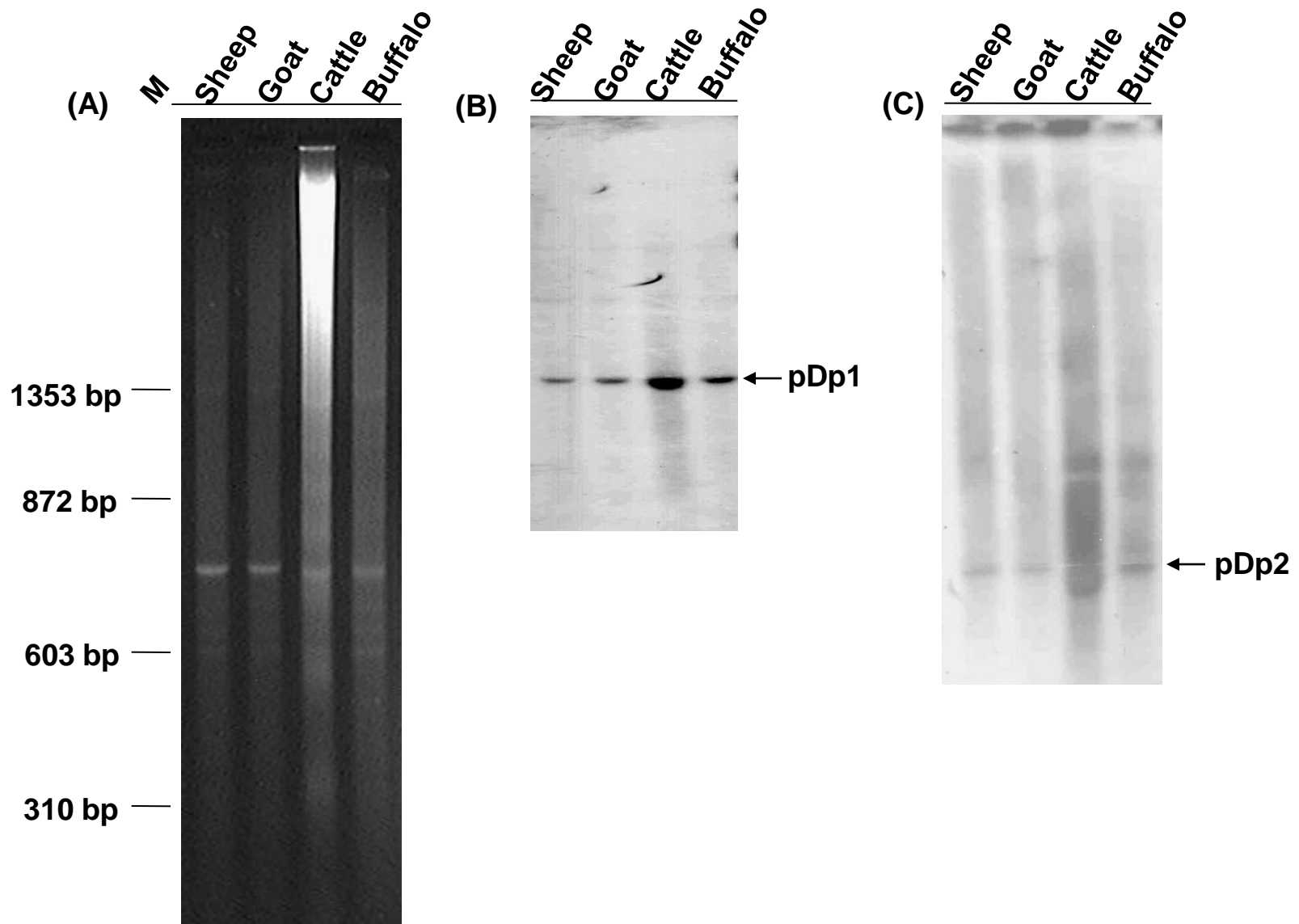

Supplement: Additional file 5 — Details of Southern blot hybridization across bovids. Representative blots showing distribution of pDp1 (A) and pDp2 (B) in buffalo, cattle, goat and sheep genome by Southern blot hybridization. Note discernible bands of 1331 and 652 bp in these species. [file 1471-2164-12-338-S5.PDF]

**(A)** pDp1

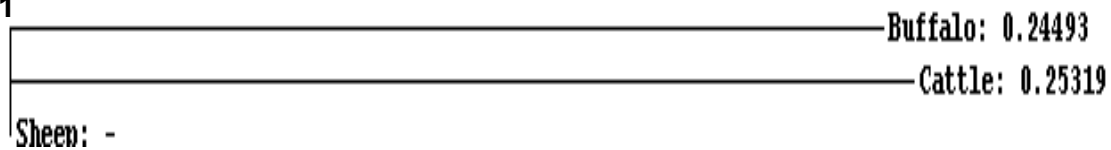

**(B)** pDp2

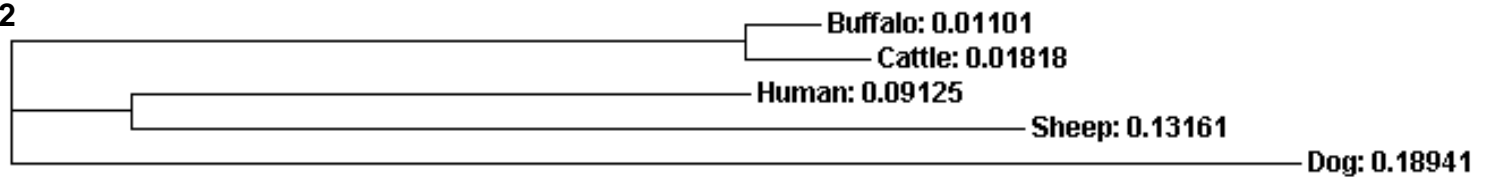

**(C)** pDp3

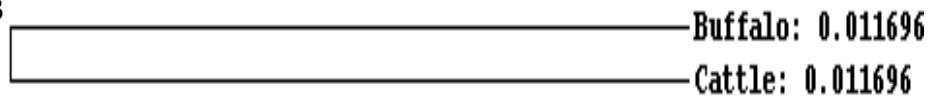

**(D)** pDp4

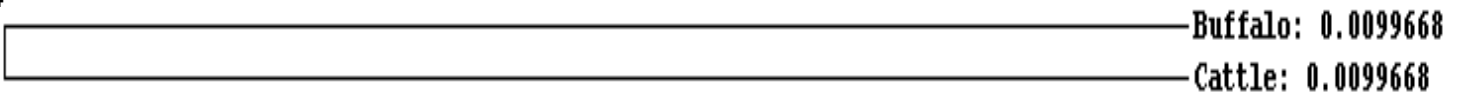

## Additional File 7

Supplement: Additional file 7 — Phylogenetic analysis. Phylogram based on percent identity of pDp1, pDp2, pDp3 and pDp4 (A-D) sequence in different species showing close relationship of buffalo with cattle. [file 1471-2164-12-338-S7.PDF]

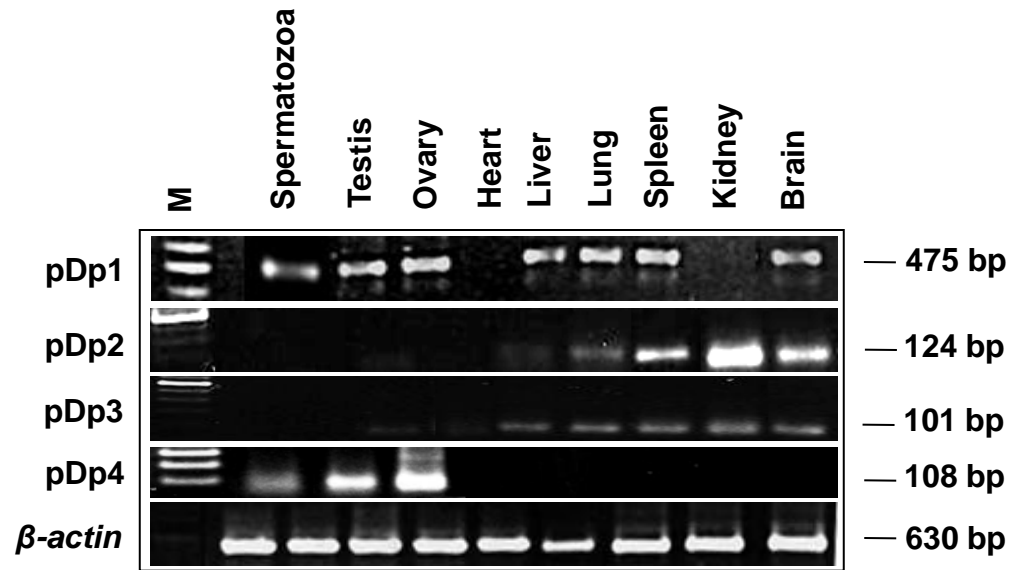

## Additional File 8

Supplement: Additional file 8 — Details of RT PCR. RT-PCR analysis of RsaI repeat sequences using internal primers and cDNA from different somatic tissues and spermatozoa of buffalo, Sequence IDs are indicated on the left and tissues are mentioned on top of the lanes. β-actin was used a positive control. M denotes 100 base pair marker. [file 1471-2164-12-338-S8.PDF]

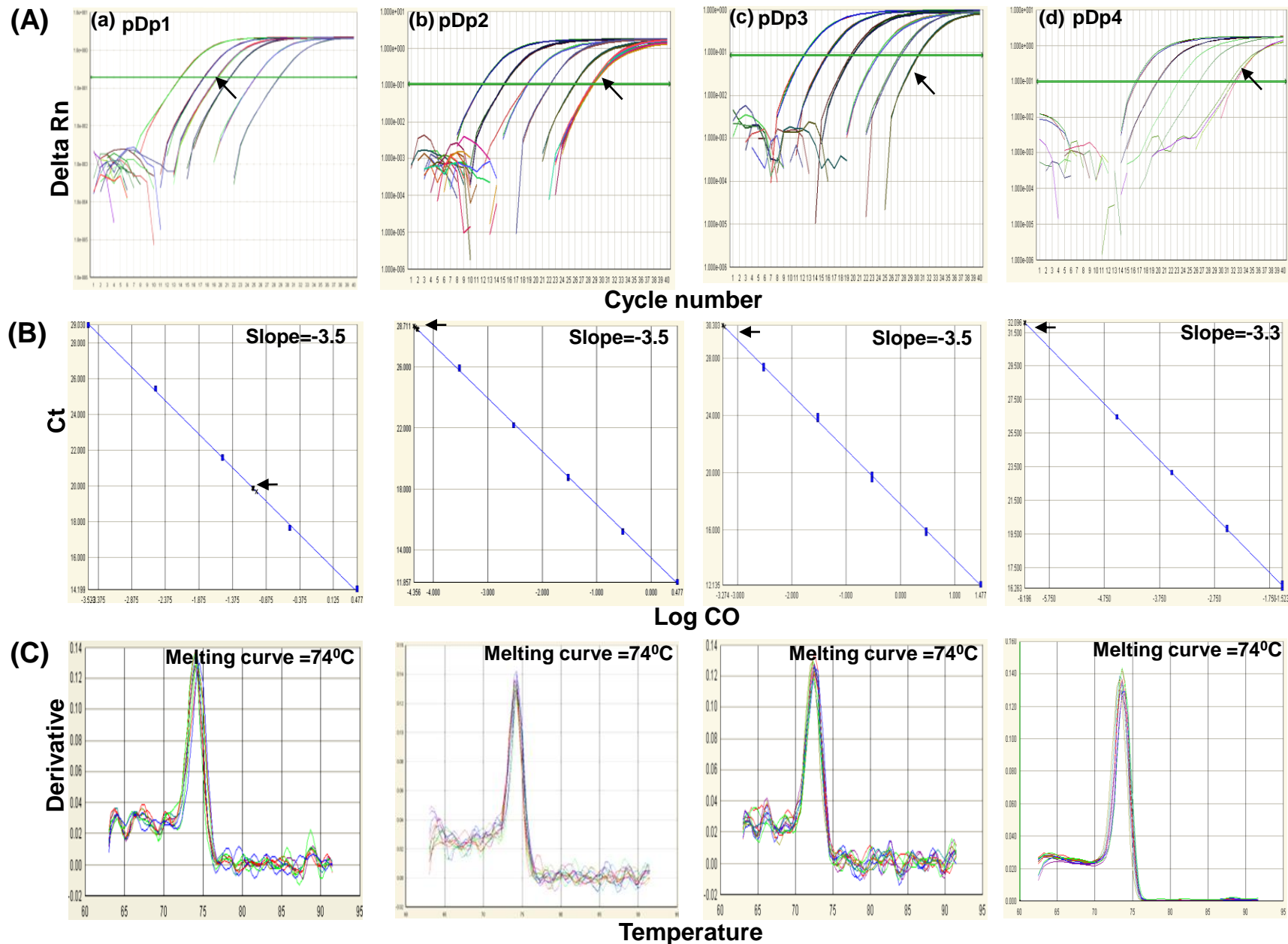

Supplement: Additional file 9 — Details of copy number calculation with Real time PCR. Standard curve based on 10 fold dilution series of pDp1, pDp2, pDp3, pDp4 and genomic DNA from buffalo, cattle, goat and sheep showing the amplification plot (a-d) panel (A), corresponding slopes of -3.3 to -3.5, panel (B) and a single dissociation peak, panel (C), substantiating maximum efficiency of the PCR reaction and high specificity of the primers with target DNA. Arrow indicates genomic DNA from buffalo, cattle, goat and sheep. [file 1471-2164-12-338-S9.PDF]

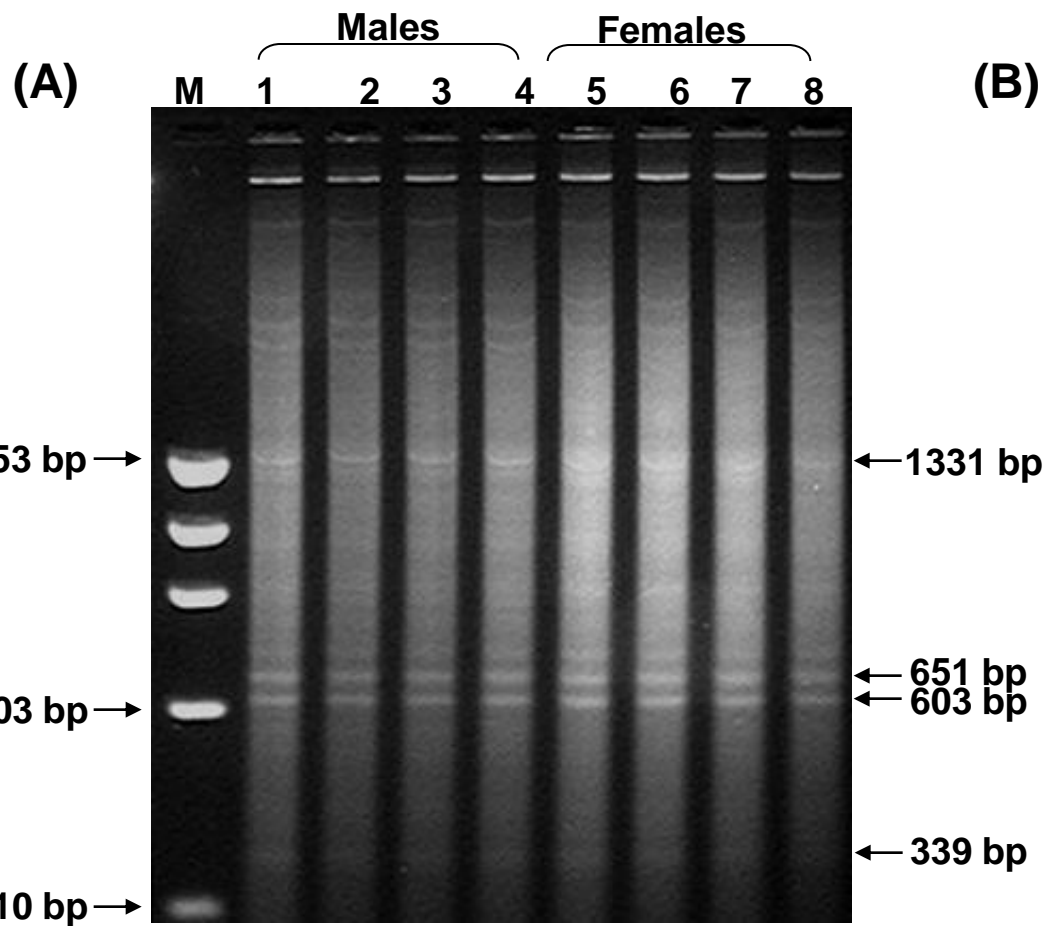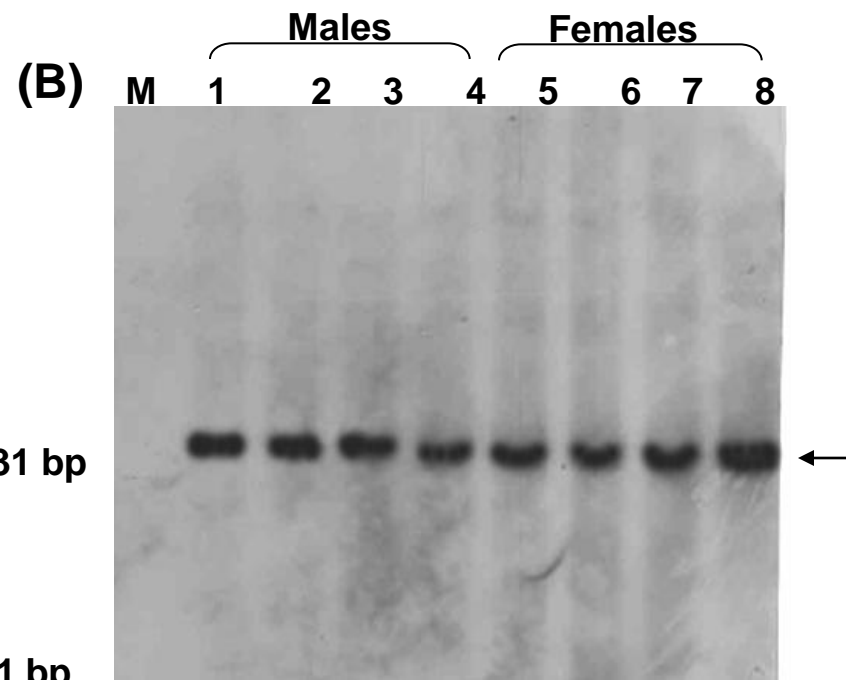

Supplement: Additional file 10 — Southern hybridization with pDp1 clone. Southern hybridization of Bubalus bubalis RsaI digested genomic DNA with pDp1 clone (A). The strongest isomorphic band corresponds to 1331 bp, indicated by an arrow (B). [file 1471-2164-12-338-S10.PDF]
