# Supplementary material for: RsaI repetitive DNA in Buffalo Bubalus bubalis representing retrotransposons, conserved in bovids, are part of the functional genes
Source: BMC Genomics. 2011 Jul 1;12:338. doi: 10.1186/1471-2164-12-338 (PMC3149587; doi:10.1186/1471-2164-12-338)
Supplement: Additional file 6 — Details of pDp1 alignment across the species. ClustalW nucleotide alignment of buffalo pDp1, 489 bp ORF sequence, with cattle, goat and sheep sequences. Note the close sequence homology among the bovids. [file 1471-2164-12-338-S6.PDF]

```
Buffalo         TCCTTTTCCTATTTGAAAGCAGTCTGTTGTTCCATGTCCAGTTCTAACTGTTGCTTCCTG 60
Cattle          TCCTTTTCCTGTTTGGAACCAGTCTGTTGTTCCATGTCCAGTTCTAACTGTTGTTTCCTG 60
Sheep           TCCTTTTCCTGTTTGGAATCAGTCTGTTGTTCCATGTCCAACTCTAACTGTTGCTTCCTG 60
Goat            TCCTTTTCCTATTTGGAACCAGTCTGTTGTTCCATGTCCAGTTCTAACTGTTGCCTCCTG 60
                ********** **** ** *********************  ***********  *****

Buffalo         ACCTGCATACAAATTTCTCAAGAGGCAGATCAGGTGGGCTGGTATTCCCATCTCTTTC-- 118
Cattle          ACCTGCATACAAATTTCTCAAGAGGCAGGTCAGGTGGTCTGGTATTCCCATCTCTTGA-- 118
Sheep           ACCCACATACAGATTTCTCAAGAGGCAGGTTAGGTGCTCTGGTATGCCTATCTCTTTC-- 118
Goat            ACCTGCATACAGATTCCTCAAGAGGCAGGTTAGGTGGTCTGGTATTCCCCACCCNTTNCT 120
                ***  ****** *** ************ * *****  ******* **   * * *

Buffalo         -AGAATTTTCCACAGTTTATTGTAATCCA-GACAGTCAAAGACTTTGGCATAGTCAGTAA 176
Cattle          -AGAATTTTCCACAGTTCGTTGTGATCCG-CACAGTCAAAGGCTTTGGCATAGTCAATAA 176
Sheep           -AGAATTTTCCACAGTTTATTGTGATCCA-CACAGTCAAAGGCTTTGGCATAGTCAATAA 176
Goat            TNANATTTTCCACAATTTATTGTGATCTAACACAGTCAAAGGCTTTGGCATAGTCAATAA 180
                    ********** **  **** ***    ********** ************** ***

Buffalo         AGCAGAAATATATGCTTTTCTGGAACTCTCTTGCTTTTTCAATGATCCAGAGGATGTTGG 236
Cattle          AGCAGAAATAGATGTTTTTCTGGAACTCTCTTGCTTTTTCCATGATCCAGCGGATGTGGG 236
Sheep           AGCAGAAATAGATGTTTTTCTGGAACTCTCTTGCTTTTTCCATGATCCAGCAGATGTTGG 236
Goat            AGCAGAAATAAATGTTTTTCTGGAACTCTCTTGCTTTTTTGATGATCCAGAGAATGTTGG 240
                ********** *** ************************  *********   **** **

Buffalo         CAATTTGATCTCTGGTTCCTCTGCCTTTTCTAAAACCAGCTTGAACATCAGGAAGTTCAC 296
Cattle          CAATTTGATCTCTGGTTCCTCTGCCTTTTCTAAAACCAGCTTGAACATCAGGAAGTTCAC 296
Sheep           CAATTTGATCTCTGGTTCCTCTGGCTTTTCTAAAACCAGCTTGAACATCAGGGAGTTCAC 296
Goat            CAATTTGATCTCTGGTTCCTCTGCCTTTTCTAAAACCAGCTTGAACATCAGGGAGTTCAC 300
                *********************** **************************** *******

Buffalo         GGTTCACGTATTGTTGAAGCCTGGCTTGGAGAATTTTGAGCATCACTTTACCAGCGTGTG 356
Cattle          GGTTCACATATTGCTGAAGCCTGGCTTGGAGAATTTTGAGCATTACTTTACTAGTGTGTG 356
Sheep           GGTTCACGTATTGCTAAAGCCTGGCTTGGAGAATTTTGAGCATTACTTTACTAGCATGTG 356
Goat            AGTTCATGTATTGCTGAAGCCCGGCTTGGAGAATTTTGAGCACTACTTTACTACCGTGTG 360
                 *****  ***** * ***** ********************  ******* *   ****

Buffalo         AGATGAGTGTAACT-GTGTGGTAGTCTGAGCGTTCTTTGGCATTGCCTTTCTTTGGGATT 415
Cattle          AGATGAGTGCAATT-GTGGGGTAGTTTGAGCATTCTTTGGCATTGCCTTTCTTTGGGATT 415
Sheep           AGATGAGTGCAAAT-GTGTGGTAGTCTGAGCATTCTTTGGCATTGCCTTTCTTTGGGATT 415
Goat            AGATGAGTGCAATTTGTGCAGTAGTTTGAGCATTCTTTGGCATTGCCTTTCTTTGGAATT 420
                ********* ** * ***  ***** ***** ************************ ***

Buffalo         AGAATGAAAAGTGACCTTTTCC-AGTCCTGTGGCCACTGCTGAGTTTTCCAAATTTTCTG 474
Cattle          GGAATGAAAACTGACCTTTTCCCAGTGCTGTGGCCACTGCTGAGTTTTCCAAATTTGCTG 475
Sheep           GGAATGAAAACTGACCTTTTCC-AGTCCTGTGGCCACTGCTGAGTTTTCCAAATTTGCTG 474
Goat            GGAATGAAAACTGACCTTTTCT-AGTCCTGTGGCCACTGCTGAGTTTTCCAAATTTGCTG 479
                 ********* **********  *** ***************************** ***

Buffalo         GCATATTGAGTGCAG 489
Cattle          GCATATTGAGTGCAG 490
Sheep           GCATATTGAGTGCAG 489
Goat            GCGTATTGAGTGCAG 494
                ** ************
```

# Additional File 6
