# Supplementary material for: RsaI repetitive DNA in Buffalo Bubalus bubalis representing retrotransposons, conserved in bovids, are part of the functional genes
Source: BMC Genomics. 2011 Jul 1;12:338. doi: 10.1186/1471-2164-12-338 (PMC3149587; doi:10.1186/1471-2164-12-338)
Supplement: Additional file 11 — Status of Exons in ACOT11 gene. Pictorial representation showing Bos taurus (A) and Bubalus bubalis ACOT11 gene (B) with their representative exons. Nucleotide position 730 to 1331 indicates region of pDp1 showing 92% homology to Bos taurus ACOT11. Full length sequence of Bubalus bubalis ACOT11 gene lacking poly A tail and exons are given in (C). [file 1471-2164-12-338-S11.PDF]

(A)

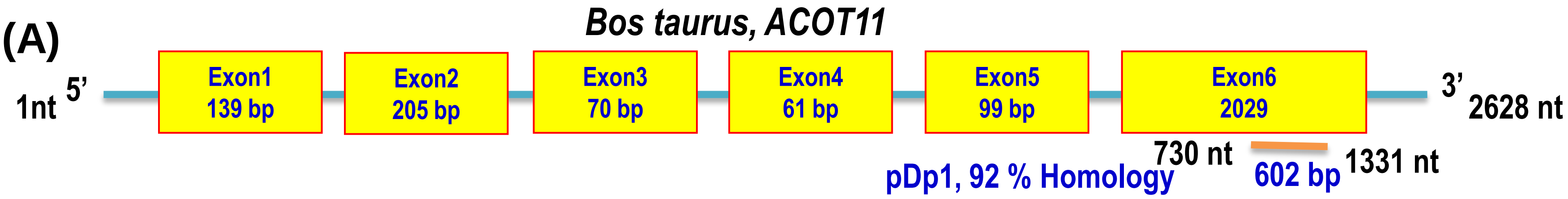

(B)

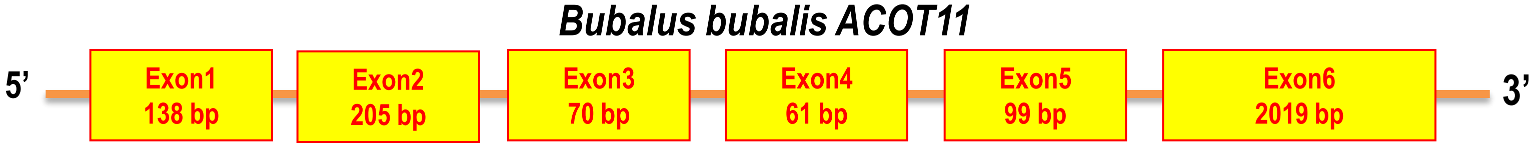

(C)

EXON1 : TGGGAGGCGAGGCGATCCCTGCGTTCGGAGTTGGAGCCGGCTGTCAGAGTCTCTGACCAGTGTCTCTCCGAGGAGGGTGCGACCAGCTAACGGGCCTCCGC
AGCAATGATCCAGACTGTTGGAAATCACCTACGAAGG
EXON2 : GGCCTGGCCTCCGTGTTCTCCAACCGCACGTCCCGGAAGTCGGCCTCACGCACTGACAGCGACAACATGGCAGATGGCGAGGGCTACCGGAACCCCACGGA
GGTGCAGATGAGCCAGCTGGTGCTGCCCTGCCACACCAACCAGCGTGGCGAGCTGAGCGTCGGGCAGCTGCTCAAGTGGATCGACGCCACCGCCTGCCTGTCCG
EXON3 : CGGAGAGGCATGCAGGGTGCCCCTGTGTCACAGCCTCCATGGATGACATCTATTTTGAGCACACCATTAG
EXON4 : TGTTGGACAAGTTGTGAATATCAAGGCCAAGGTGAACCGAGCCTTCAACTCTAGCATGGAG
EXON5 : GTGGGCATCCAGGTGGTCTCCGAGGACCTGTGCTCTGAGAAGCAGTGGTGTGTGTGCAAGGCCTTAGCCACCTTCGTGGCCCACAGGGAGCTCTCCAAG
EXON6 : GTGAAGCTGAAGCCAACTGTGCCTCGGACCGAAGAGGAAAAGACGGAGCACAGTGTGGCGGCCGAGCGCCGGCGCATGCGACTGGTCTACGCAGACACCAT
TAAGGACCTCCTCGCCAACTGCGTCATTCAGGATGGTGAGCGTCATGGGTAGGGAGGGCAGCTCGTGTCCTACATGCTCCCCTCTGTCTGGGGCTCCTACTGGGCAG
AGAAAGGAAGGAAGTCTCCCTGGAATATCCACCCAGTGATGGCCTCAGGCCTGGATAACACTCTTGCCTCAAGGTCCCTTAGTGGGGAAAAGGGAGCATTTGGACCT
CTCGCCCAGCTCCCAGGAGCCATGAAGATGAAGCTGGGACACATAGAAAACAGTTTGTCATCCACTAAATGCATTTGGGTCTCTAGGAGTCAAGGTATTTGGGAAGG
TAGTATTGAGCTTGGCCTTGAGGACCCTCAGTTCAGTCGCTCAGTCATGTGCAACTCTTTGCGACCCCATGAATTGCAGCACGCCAGGCCTCCCTGTCCATCACCAG
CTCCTGGAGTTCACCCAAACTCATGTCCATCGAGTTGGTGATGCCATCCAGCCATCTCATCCTCTGTTGTTCCCTTCTCCTCCTGCCCCTAATCGAGCCGGCTGTCA
GAGTCTTTTCCAATGAGTCAACTTTTCGCATGAGGTGGCCAAAGTACTGGAGTTTCAGCTTTAGCATCATTCCTTCCAAAGAAATCCCAGGGCTGATCTCCTTCAGA
ATGGACTGGTTGGATCTCCTTTCAGTCCAAGGGACTCTCAAGAGTCTTCTCCAACACCACAGTTCAAAGGCATCAATTCTTCGGCGCTCAGCCTTCTTCACAGTCCA
ACTCTCACGTCCATACATGACCACAGGAAAAACCATAGCCTTGACTAGACGAACCTTTGTTGGCAAAGTAATGTCTCTGCTTTTTAGCATGCTATCTAGGTTGGTCA
TAACTTTCCTTCCAAGGAGGAGTAAGCGTCTTTCATTCCATGGGCTGCAGTCACCATCTGCAGTGATTTTGGAGCCCCCAAAAATAAAAGTCTGACACTGTTTTCC
CCGTCTATTTCCCATGAAGTGATGGGACCGGGATGCCATGATCTTCGTTTTCTGAATGCTGAGCTTTAAGCCAACTTTTTCACTCTCCACTTTCACCTTCATCAATA
GGCTTTTGAGTTCCTCTTCACTTTCTGCCATAAGGGTGGTGTCATCTGCATATCTGAGGTTATTGATATTTCTCCCAGCAATCTTGGTTCCAGCTTGCGTTTCTTCC
AGTCCAGTGTTTCTCATGATGTACTCTGCATATAAGTTAAATAAACAGGGTGACAATATACAGCCTTGACGAACTCCTTTTCCTATTTGGAACCAGTCTGTTGTTCC
ATGTCCAGTTCTAACTGTTGCTTCCTGACCTGCATACAGATTTCTCAAGAGGCAGATCAGGTGGTCTGGTATTCCCATCTCTTTCAGAATTTTCCACAGTTTCTTGT
AATCCACACAGTCAAAGGCTTTGGCATAGTCAATAAAGCAGAAATAGATGCTTTTCTGGAACTCTCTTGCTTTTTCCATGATCCAGAGGATGTTGGCAATTTGATCT
CTGGTTCCTCTGCCTTTTCTAAAACCAGCTTGAACATCAGGAAGTTCACGGTTCACGTATTGCTGAAGCCTGGCTTGGAGAATTTTGAGCATTACTTTACTAGCGTG
TGAGATGAGTGCAATTGTGTGGTAGTTTGAGCATTCTTTGGCATTGCCTTTCTTTGGGATTGGAATGAAAACTGACCTTTTCCAGTCCTGTGGCCACTGCTGAGTTT
TCCAAATGTGCTGGCATATTGAGTGCAGCACTTTCACAGCATCATCTTTCAGGATTTGAAATAGCTCACTGGAATTCCATCACCTCCACTAGCTTTGTTTATAGTGA
TGCTTTCTAAGCCCACTTGACTTCACATTCCAGGTTGTCTGGCTCTAGGTGAGTGATCACACCATCGTGATTATCTTGGTCATGAAGATCTTTTTAGGA

Additional File 11
