# Supplementary material for: RsaI repetitive DNA in Buffalo Bubalus bubalis representing retrotransposons, conserved in bovids, are part of the functional genes
Source: BMC Genomics. 2011 Jul 1;12:338. doi: 10.1186/1471-2164-12-338 (PMC3149587; doi:10.1186/1471-2164-12-338)
Supplement: Additional file 12 — ClustalW alignment of ACOT11 gene. ClustalW alignment of buffalo ACOT11 gene with cattle (NM_001103275.1). Note the high level of sequence homology (92%) between the two species. [file 1471-2164-12-338-S12.PDF]

```
BTACOT11        TGGGAGGCGAGGCGATCCCTGCGTTCGGAGTTGGAGCCGGCTGTCAGAGTCTCTGACCAG 60
BBACOT11        TGGGAGGCGAGGCGATCCCTGCGTTCGGAGTTGGAGCCGGCTGTCAGAGTCTCTGACCAG 60
                ************************************************************

BTACOT11        TGTCTCTCCGAGGAGGGTGCGACCAGCTAACGGGCCTCCGCAGCAATGATCCAGACTGTT 120
BBACOT11        TGTCTCTCCGAGGAGGGTGCGACCAGCTAACGGGCCTCCGCAGCAATGATCCAGACTGTT 120
                ************************************************************

BTACOT11        GGAAATCACCTACGAAGGGGCCTGGCCTCCGTGTTCTCCAACCGCACGTCCCGGAAGTCG 180
BBACOT11        GGAAATCACCTACGAAGGGGCCTGGCCTCCGTGTTCTCCAACCGCACGTCCCGGAAGTCG 180
                ************************************************************

BTACOT11        GCCTCACGCACTGACAGCGACAACATGGCAGATGGCGAGGGCTACCGGAACCCCACGGAG 240
BBACOT11        GCCTCACGCACTGACAGCGACAACATGGCAGATGGCGAGGGCTACCGGAACCCCACGGAG 240
                ************************************************************

BTACOT11        GTGCAGATGAGCCAGCTGGTGCTGCCCTGCCACACCAACCAGCGTGGCGAGCTGAGCGTC 300
BBACOT11        GTGCAGATGAGCCAGCTGGTGCTGCCCTGCCACACCAACCAGCGTGGCGAGCTGAGCGTC 300
                ************************************************************

BTACOT11        GGGCAGCTGCTCAAGTGGATCGACGCCACCGCCTGCCTGTCCGCGGAGAGGCATGCAGGG 360
BBACOT11        GGGCAGCTGCTCAAGTGGATCGACGCCACCGCCTGCCTGTCCGCGGAGAGGCATGCAGGG 360
                ************************************************************

BTACOT11        TGCCCCTGTGTCACAGCCTCCATGGATGACATCTATTTTGAGCACACCATTAGTGTTGGA 420
BBACOT11        TGCCCCTGTGTCACAGCCTCCATGGATGACATCTATTTTGAGCACACCATTAGTGTTGGA 420
                ************************************************************

BTACOT11        CAAGTTGTGAATATCAAGGCCAAGGTGAACCGAGCCTTCAACTCTAGCATGGAGGTGGGC 480
BBACOT11        CAAGTTGTGAATATCAAGGCCAAGGTGAACCGAGCCTTCAACTCTAGCATGGAGGTGGGC 480
                ************************************************************

BTACOT11        ATCCAGGTGGTCTCCGAGGACCTGTGCTCTGAGAAGCAGTGGTGTGTGTGCAAGGCCTTA 540
BBACOT11        ATCCAGGTGGTCTCCGAGGACCTGTGCTCTGAGAAGCAGTGGTGTGTGTGCAAGGCCTTA 540
                ************************************************************

BTACOT11        GCCACCTTCGTGGCCCACAGGGAGCTCTCCAAGGTGAAGCTGAAGCCAACTGTGCCTCGG 600
BBACOT11        GCCACCTTCGTGGCCCACAGGGAGCTCTCCAAGGTGAAGCTGAAGCCAACTGTGCCTCGG 600
                ************************************************************

BTACOT11        ACCGAAGAGGAAAAGACGGAGCACAGTGTGGCGGCCGAGCGCCGGCGCATGCGACTGGTC 660
BBACOT11        ACCGAAGAGGAAAAGACGGAGCACAGTGTGGCGGCCGAGCGCCGGCGCATGCGACTGGTC 660
                ************************************************************

BTACOT11        TACGCAGACACCATTAAGGACCTCCTCGCCAACTGCGTCATTCAGGATGGTGAGCGTCAT 720
BBACOT11        TACGCAGACACCATTAAGGACCTCCTCGCCAACTGCGTCATTCAGGATGGTGAGCGTCAT 720
                ************************************************************

BTACOT11        GGGTAGGGAGGGCAGCTCGTGTCCTACATGCTCCCCTCTGTCTGGGGCTCCTACTGGGCA 780
BBACOT11        GGGTAGGGAGGGCAGCTCGTGTCCTACATGCTCCCCTCTGTCTGGGGCTCCTACTGGGCA 780
                ************************************************************

BTACOT11        GAGAAAGGAAGGAAGTCTCCCTGGAATATCCACCCAGTGATGGCCTCAGGCCTGGATAAC 840
BBACOT11        GAGAAAGGAAGGAAGTCTCCCTGGAATATCCACCCAGTGATGGCCTCAGGCCTGGATAAC 840
                ************************************************************

BTACOT11        ACTCTTGCCTCAAGGTCCCTTAGTGGGGAAAAGGGAGCATTTGGACCTCTCGCCCAGCTC 900
BBACOT11        ACTCTTGCCTCAAGGTCCCTTAGTGGGGAAAAGGGAGCATTTGGACCTCTCGCCCAGCTC 900
                ************************************************************

BTACOT11        CCAGGAGCCATGAAGATGAAGCTGGGACACATAGAAAACAGTTTGTCATCCACTAAATGC 960
BBACOT11        CCAGGAGCCATGAAGATGAAGCTGGGACACATAGAAAACAGTTTGTCATCCACTAAATGC 960
                ************************************************************
```

```
BTACOT11        ATTTGGGTCTCTAGGAGTCAAGGTATTTGGGAAGGTAGTATTGAGCTTGGCCTTGAGGAC 1020
BBACOT11        ATTTGGGTCTCTAGGAGTCAAGGTATTTGGGAAGGTAGTATTGAGCTTGGCCTTGAGGAC 1020
                ************************************************************

BTACOT11        CCTCAGTTCAGTCGCTCAGTCATGTGCAACTCTTTGCGACCCCATGAATTGCAGCACGCC 1080
BBACOT11        CCTCAGTTCAGTCGCTCAGTCATGTGCAACTCTTTGCGACCCCATGAATTGCAGCACGCC 1080
                ************************************************************

BTACOT11        AGGCCTCCCTGTCCATCACCAGCTCCTGGAGTTCACCCAAACTCATGTCCATCGAGTTGG 1140
BBACOT11        AGGCCTCCCTGTCCATCACCAGCTCCTGGAGTTCACCCAAACTCATGTCCATCGAGTTGG 1140
                ************************************************************

BTACOT11        TGATGCCATCCAGCCATCTCATCCTCTGTTGTTCCCTTCTCCTCCTGCCCCTAATCTCTC 1200
BBACOT11        TGATGCCATCCAGCCATCTCATCCTCTGTTGTTCCCTTCTCCTCCTGCCCCTAATCGAGC 1200
                ********************************************************   *

BTACOT11        CCAGCATCAGAGTCTTTTCCAATGAGTCAACTCTTCCCATGAGATGGCCAAAGTATTAGA 1260
BBACOT11        CGGCTGTCAGAGTCTTTTCCAATGAGTCAACTTTTCGCATGAGGTGGCCAAAGTACTGGA 1260
                *     ************************** *** ****** *********** * **

BTACOT11        GTTTCAGCTTTAGCATCAGTCCTTCCAAAGAACACCCAGGACTGATCTCCTTCAGAATGG 1320
BBACOT11        GTTTCAGCTTTAGCATCATTCCTTCCAAAGAAATCCCAGGGCTGATCTCCTTCAGAATGG 1320
                ****************** *************  ****** *******************

BTACOT11        ACTGGTTGGATCTCCTTGCAGTCCAAGGGACTCTCAAGAGTCTTCTCCAACACTACAGTT 1380
BBACOT11        ACTGGTTGGATCTCCTTTCAGTCCAAGGGACTCTCAAGAGTCTTCTCCAACACCACAGTT 1380
                ***************** *********************************** ******

BTACOT11        CAAAAGCATCAATTCTTCGGCACTCAGCCTTCTTCACAGTCCAACTCTCACATCCATACA 1440
BBACOT11        CAAAGGCATCAATTCTTCGGCGCTCAGCCTTCTTCACAGTCCAACTCTCACGTCCATACA 1440
                **** **************** ***************************** ********

BTACOT11        TGACCACAGGAAAAACCATAGCCTTGACTAGACGGACCTTTGTTGGCAAAGTAATGTCTC 1500
BBACOT11        TGACCACAGGAAAAACCATAGCCTTGACTAGACGAACCTTTGTTGGCAAAGTAATGTCTC 1500
                ********************************** *************************

BTACOT11        TGCTTTTGAATATGCTATCTAGGTTGGTCATAACTTTCCTTCCAAGGAA---TAAGCATC 1557
BBACOT11        TGCTTTTTAGCATGCTATCTAGGTTGGTCATAACTTTCCTTCCAAGGAGGAGTAAGCGTC 1560
                ******* *  *************************************    ***** **

BTACOT11        TTTTAATTTCATGGCTGCAATCACCATCTGCAGTGATTTTGGAGCCCC--AAAAAATAAA 1615
BBACOT11        TTTCATTCCATGGGCTGCAGTCACCATCTGCAGTGATTTTGGAGCCCCCCAAAAATAAAA 1620
                *** * *     ******* ****************************  *****  ***

BTACOT11        GTCTGACACTGTTTCCACTGTTTCCCCATCTATTTGCTATGAAGTGATGGGACCAG-ATG 1674
BBACOT11        GTCTGACACTGTTT--------TCCCCGTCTATTTCCCATGAAGTGATGGGACCGGGATG 1672
                **************        ***** ******* * **************** * ***

BTACOT11        CCATGATCTTCGTTTTCTGAATGTTGAGCTTTAAGCCAACTTTTTCACTCTCCTCTTTCA 1734
BBACOT11        CCATGATCTTCGTTTTCTGAATGCTGAGCTTTAAGCCAACTTTTTCACTCTCCACTTTCA 1732
                *********************** ***************************** ******

BTACOT11        CCTTCATCAAGAGGCTTTTTAGTTCCTTTTCACTTTCTGCCATAAGGGTGGTGTCATCTG 1794
BBACOT11        CCTTCATCAATAGGCTTTTGAGTTCCTCTTCACTTTCTGCCATAAGGGTGGTGTCATCTG 1792
                ********** ******** ******* ********************************

BTACOT11        CATATCTGAGGTTATTGATAGTCCTCCCGGCAATCTTTATTCCAGCTTATGCTTCCTCCA 1854
BBACOT11        CATATCTGAGGTTATTGATATTTCTCCCAGCAATCTTGGTTCCAGCTTGCGTTTCTTCCA 1852
                ******************** * ***** ********  *********  * *** ****
```

```
BTACOT11        GCCCAGCGTTTCTCATGATGTACTCTGCATATAAGTTAAATAAGCAGGGTGACAACATAC 1914
BBACOT11        GTCCAGTGTTTCTCATGATGTACTCTGCATATAAGTTAAATAAACAGGGTGACAATATAC 1912
                * **** ************************************ *********** ****

BTACOT11        AGCCTTGACGTACTCCTTTTCCTATTTGGAACCAGTCTCTTGTTCCATGTCCAGTTCTAA 1974
BBACOT11        AGCCTTGACGAACTCCTTTTCCTATTTGGAACCAGTCTGTTGTTCCATGTCCAGTTCTAA 1972
                ********** *************************** *********************

BTACOT11        CTGTTGCTTCCTGACCTGCATATAGGTTTCTCAAGAGGCAGGTCAGGTGGTCTGATATTC 2034
BBACOT11        CTGTTGCTTCCTGACCTGCATACAGATTTCTCAAGAGGCAGATCAGGTGGTCTGGTATTC 2032
                ********************** ** *************** ************ *****

BTACOT11        CCATCTCTTTCAGAATTTTCCACAGTTTCTTGTAATCCACACAGTCAAAGGCTTTGGCAT 2094
BBACOT11        CCATCTCTTTCAGAATTTTCCACAGTTTCTTGTAATCCACACAGTCAAAGGCTTTGGCAT 2092
                ************************************************************

BTACOT11        AGTCGAGAAAGCAGAAATAGATGTTTTTCTTGAACTCTCTTGCTTTTTCCATGATCCAGT 2154
BBACOT11        AGTCAATAAAGCAGAAATAGATGCTTTTCTGGAACTCTCTTGCTTTTTCCATGATCCAGA 2152
                **** * **************** ****** ****************************

BTACOT11        GGATGTTGGCAATTTCGTCTCTGGTTCCTCTGCCTTTTCTAAAACCAGTTTGAACATCAG 2214
BBACOT11        GGATGTTGGCAATTTGATCTCTGGTTCCTCTGCCTTTTCTAAAACCAGCTTGAACATCAG 2212
                ***************  ******************************* ***********

BTACOT11        GAAGTTCACGGTTCACGTATTGCTGAAGCCTGGCTTGGAGAATTTTGAGCATTACTTTAC 2274
BBACOT11        GAAGTTCACGGTTCACGTATTGCTGAAGCCTGGCTTGGAGAATTTTGAGCATTACTTTAC 2272
                ************************************************************

BTACOT11        TAGCGTGTGAGATGAGTGCAATTGTGCAGTCGTTTGAGCATTCTTTGGCATTGCCTTTCT 2334
BBACOT11        TAGCGTGTGAGATGAGTGCAATTGTGTGGTAGTTTGAGCATTCTTTGGCATTGCCTTTCT 2332
                **************************  ** *****************************

BTACOT11        TTGGGATTGAAATGAAAACTGACCTTTTCCAGTCCTGTGACTACTGCTGAGTTTTCCAAA 2394
BBACOT11        TTGGGATTGGAATGAAAACTGACCTTTTCCAGTCCTGTGGCCACTGCTGAGTTTTCCAAA 2392
                ********* ***************************** * ******************

BTACOT11        TGTGCTGGCATATTGAGTGCAGCACTTTCACAGCATCGTCATCTTCTAGGATTTGAAATA 2454
BBACOT11        TGTGCTGGCATATTGAGTGCAGCACTTTCACAGCA---TCATCTTTCAGGATTTGAAATA 2449
                ***********************************   *******  *************

BTACOT11        GTTCAACTGGAATTCCATCACCTCCACTAGCTTTGTTTGTAGTGATGCTTTCTAAGGCCT 2514
BBACOT11        GCTCA-CTGGAATTCCATCACCTCCACTAGCTTTGTTTATAGTGATGCTTTCTAAG-CCC 2507
                * *** ******************************** ***************** **

BTACOT11        ACTTGACTTCACATTCCAGGTTGTCTGGCTCTAGGTGAGTGATCACACCATCATGATTAT 2574
BBACOT11        ACTTGACTTCACATTCCAGGTTGTCTGGCTCTAGGTGAGTGATCACACCATCGTGATTAT 2567
                **************************************************** *******

BTACOT11        CTTGGTCATGAAGATCTTTTTAGGACTTAAAAAAAAAAAAAAAAAAAAAAAAAA 2628
BBACOT11        CTTGGTCATGAAGATCTTTTTAGGA----------------------------- 2592
                *************************
```

Additional File 12
